# Supplementary figures and images for: Defining the minimal components of the influenza A virus replication machinery via an in vitro reconstitution system
Source: PLoS Biol. 2023 Nov 9;21(11):e3002370. doi: 10.1371/journal.pbio.3002370 (PMC10662765; doi:10.1371/journal.pbio.3002370)

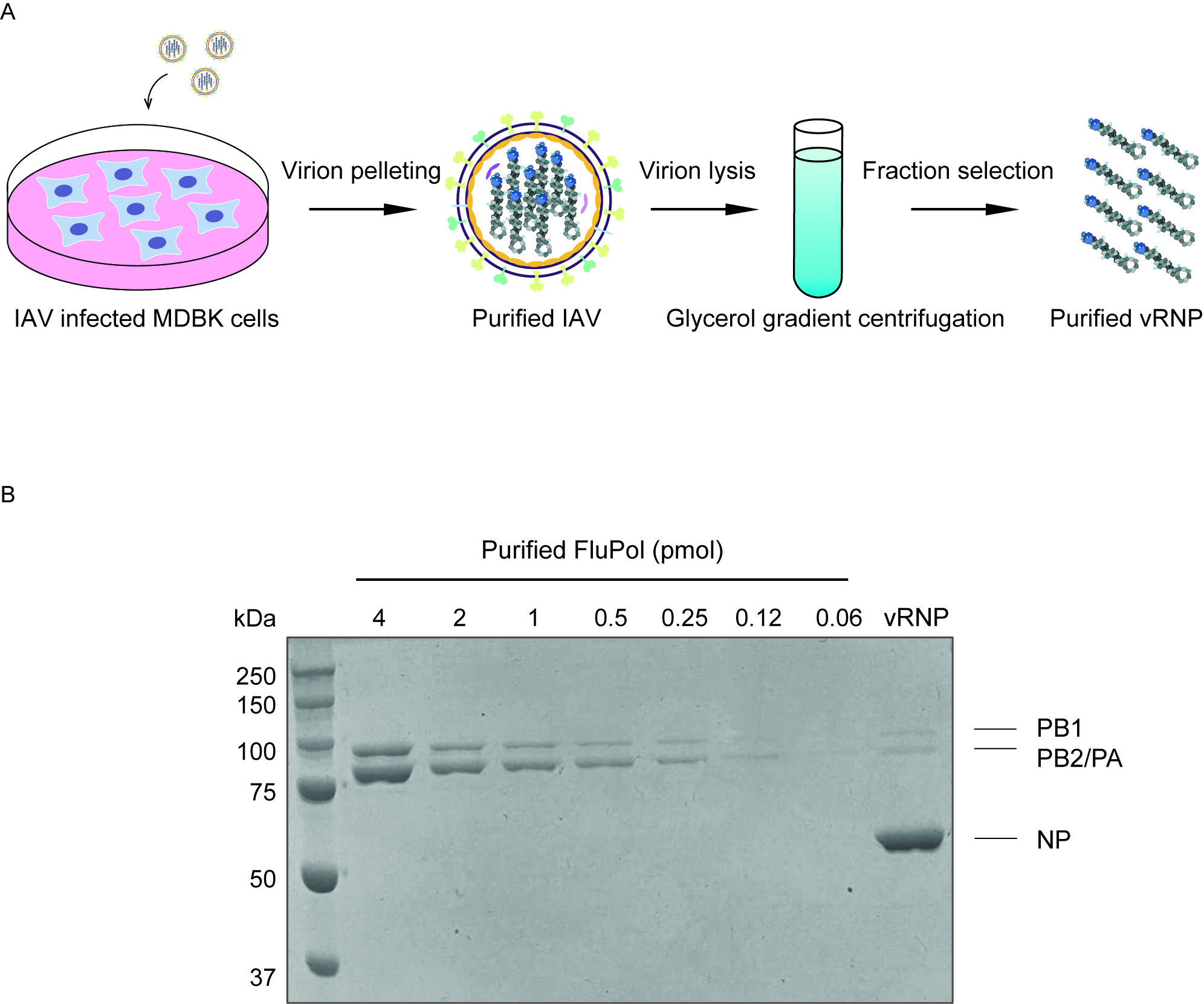

Supplement: S1 Fig — (A) Schematic illustrating the glycerol gradient purification of vRNPs isolated from virions produced in MDBK cells. (B) Estimation of vRNP molar concentration through comparison of vRNP with known concentration of viral polymerase (FluPol). Original image can be found in S1 Raw Images. (TIF) [file pbio.3002370.s001.tif]

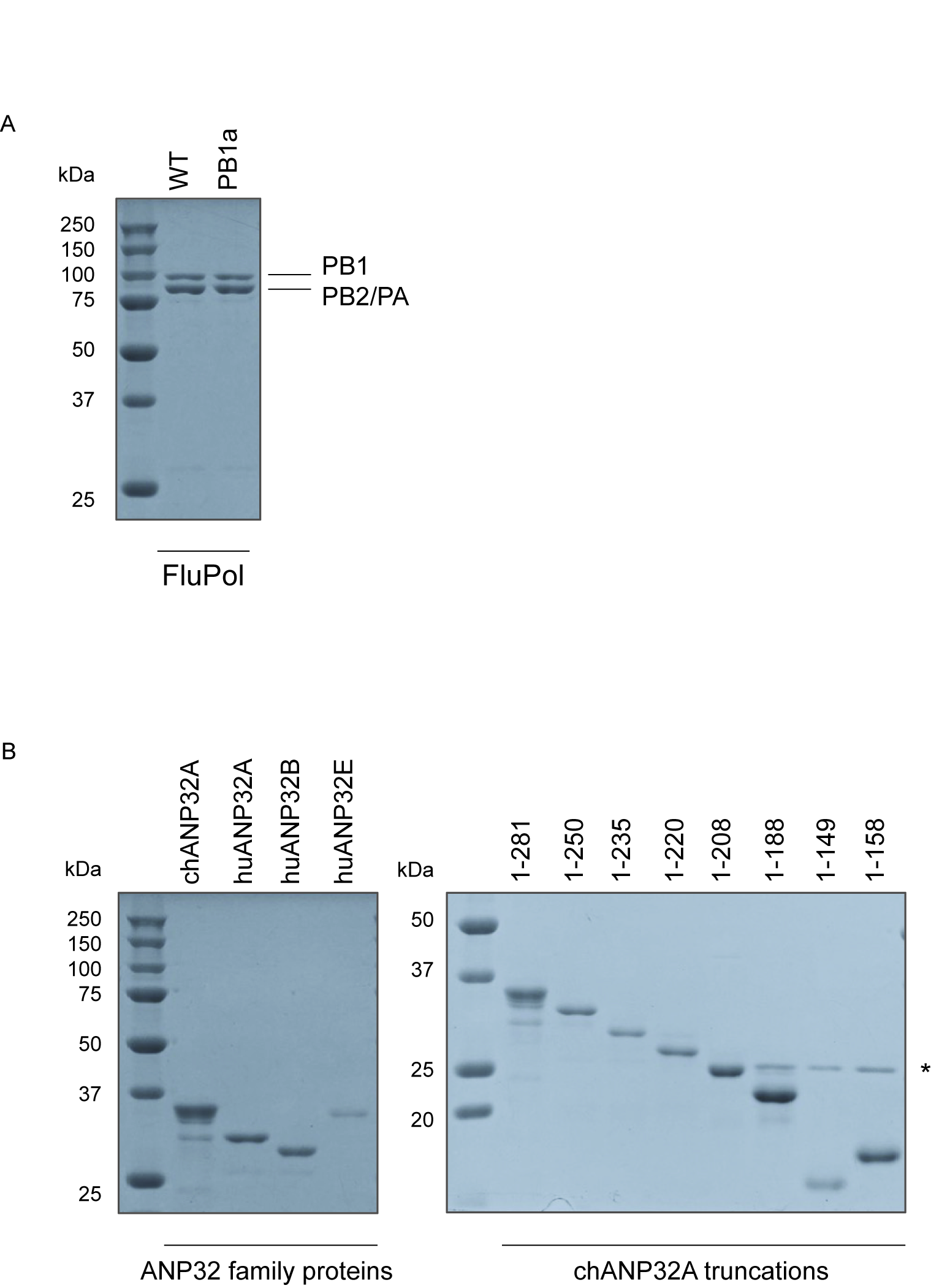

Supplement: S2 Fig — (A) WT and catalytically inactive (PB1a) FluPol purified from insect cells. (B) ANP32 family proteins including chicken ANP32A (chANP32A), human ANP32A (huANP32A), human ANP32B (huANP32B), human ANP32E (huANP32E), and C-terminally truncated chANP32A mutants purified from E. coli. * indicates residual cleaved GST tag. Original images can be found in S1 Raw Images. ANP32, acidic nuclear phosphoprotein 32; SDS-PAGE, sodium dodecyl sulfate polyacrylamide gel electrophoresis; WT, wild type. (TIF) [file pbio.3002370.s002.tif]

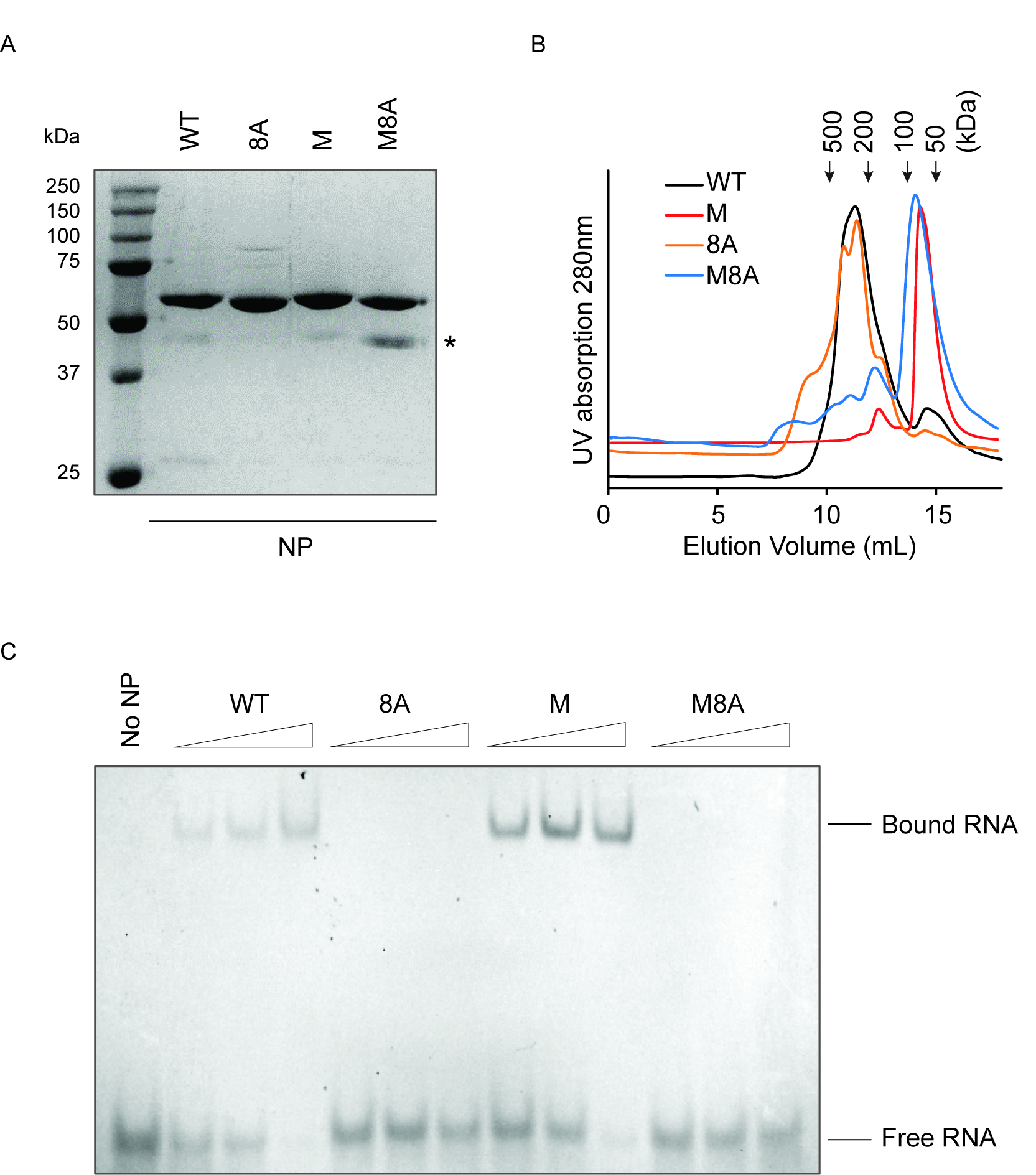

Supplement: S3 Fig — (A) WT, RNA-binding deficient mutant (8A), monomeric mutant (M), and RNA-binding deficient monomeric mutant (M8A) NP proteins were purified from E. coli. * indicates NP degradation product. (B) Size-exclusion chromatography curves of the purification of NP proteins. Size markers are shown above the curves. The y-axes were rescaled to make main peaks of equal heights. (C) RNA-binding activity of WT and mutant NP assessed by EMSA. Original images can be found in S1 Raw Images. EMSA, electrophoretic mobility shift assay; NP, nucleoprotein; SDS-PAGE, sodium dodecyl sulfate polyacrylamide gel electrophoresis; WT, wild type. (TIF) [file pbio.3002370.s003.tif]

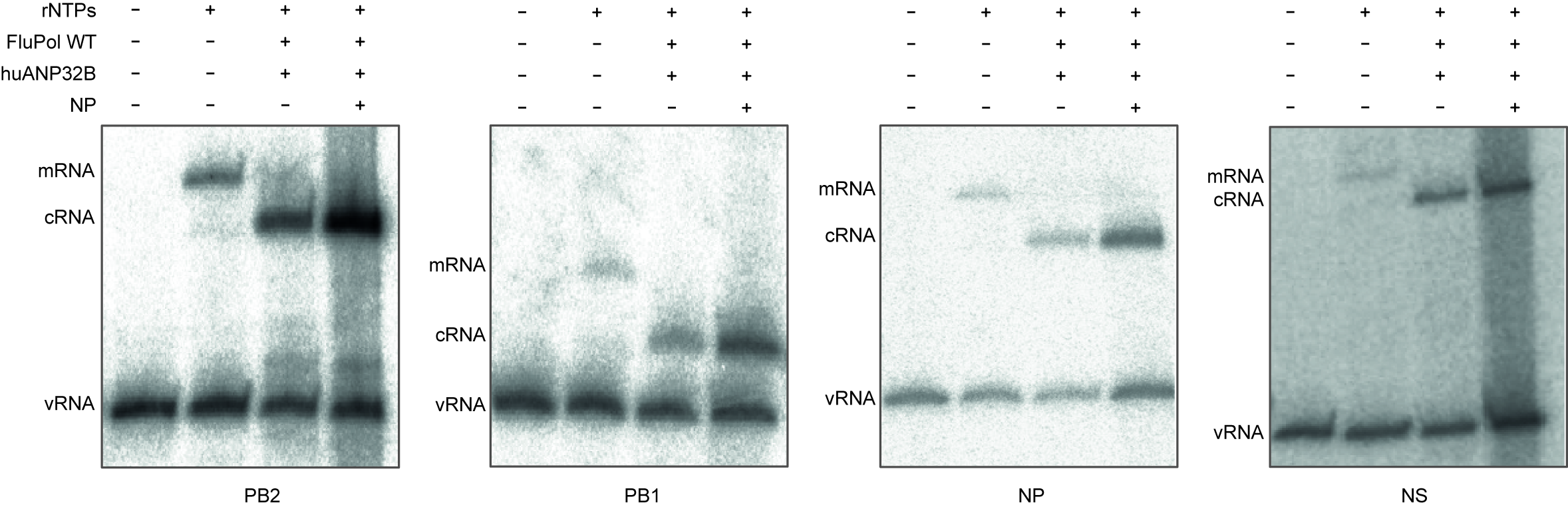

Supplement: S4 Fig — Effect of viral polymerase (FluPol), huANP32B, and NP alone or together on vRNP activity in segment 1 (PB2), segment 2 (PB1), segment 5 (NP), and segment 8 (NS), in the presence of globin mRNA as cap donor. A representative result of RNA analysis by primer extension is shown. Original images can be found in S1 Raw Images. NP, nucleoprotein; NS, nonstructural; PB1, polymerase basic 1; PB2, polymerase basic 2; vRNP, viral ribonucleoprotein. (TIF) [file pbio.3002370.s004.tif]
