# Supplementary material for: Defining the minimal components of the influenza A virus replication machinery via an in vitro reconstitution system
Source: PLoS Biol. 2023 Nov 9;21(11):e3002370. doi: 10.1371/journal.pbio.3002370 (PMC10662765; doi:10.1371/journal.pbio.3002370)
Supplement: S1 Raw Images — (PDF) [file pbio.3002370.s006.pdf]

Fig 1A repeat 1

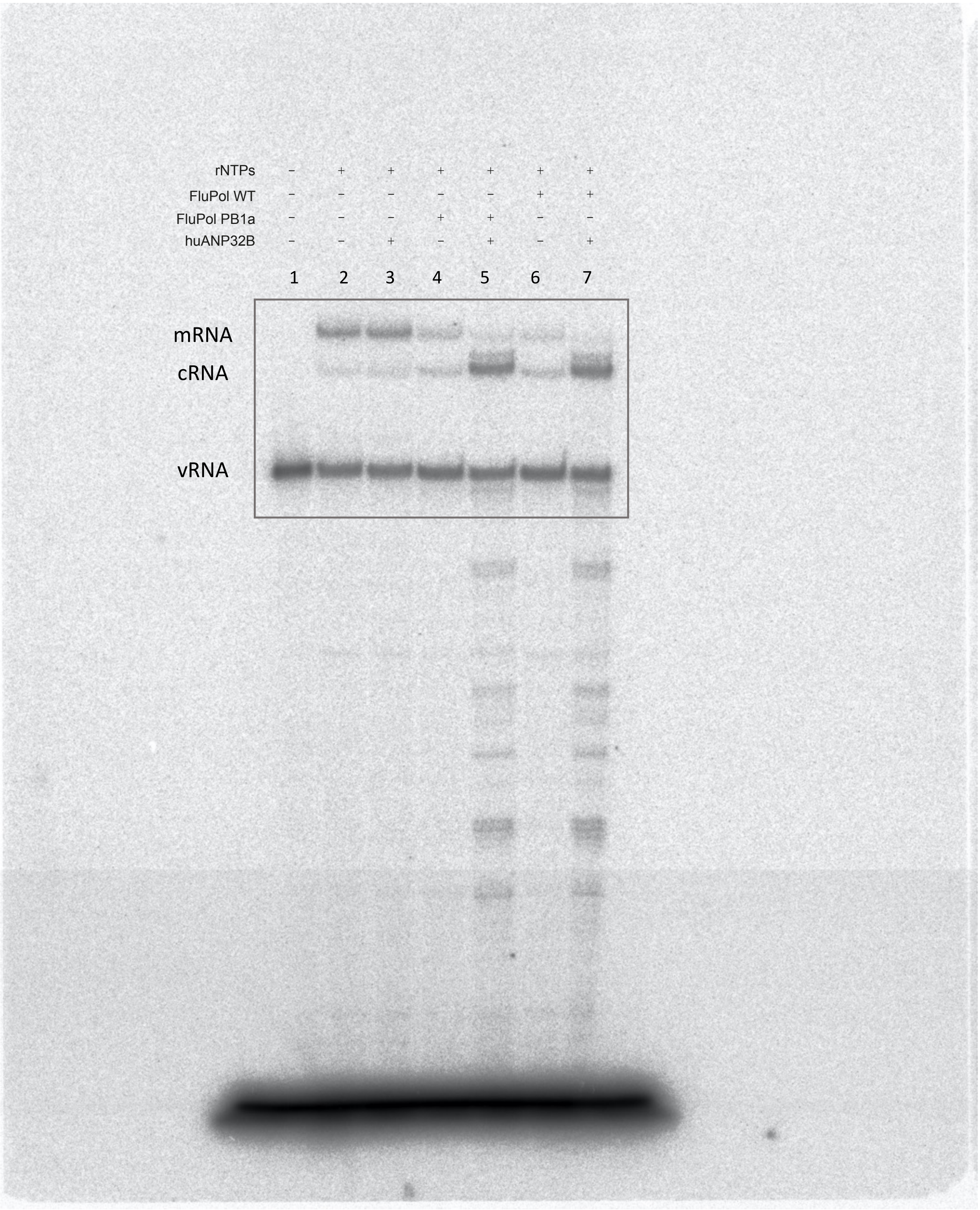

visualised by phosphorimaging on an FLA-5000 scanner

Fig 1A repeat 2

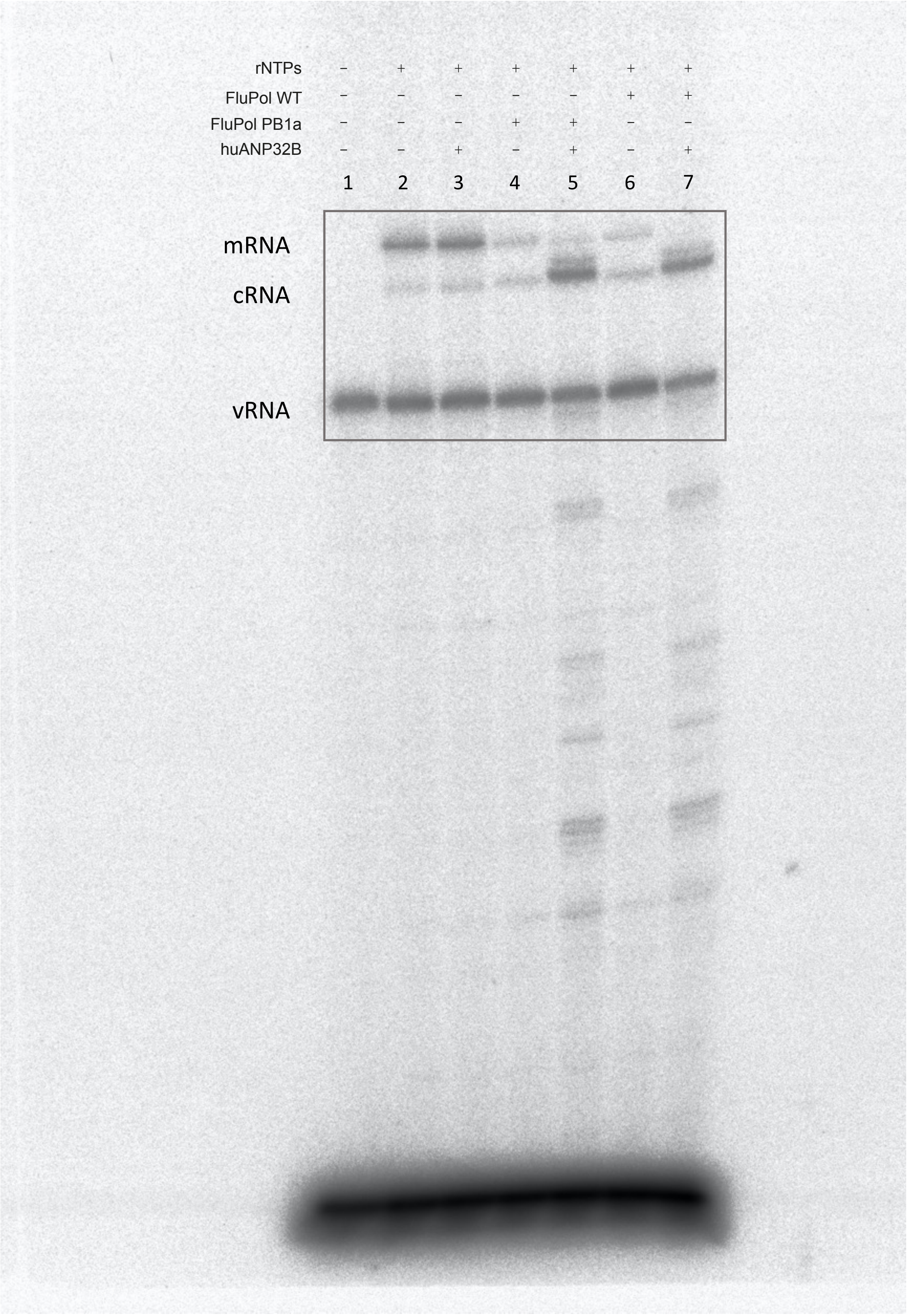

visualised by phosphorimaging on an FLA-5000 scanner

Fig 1A repeat 3

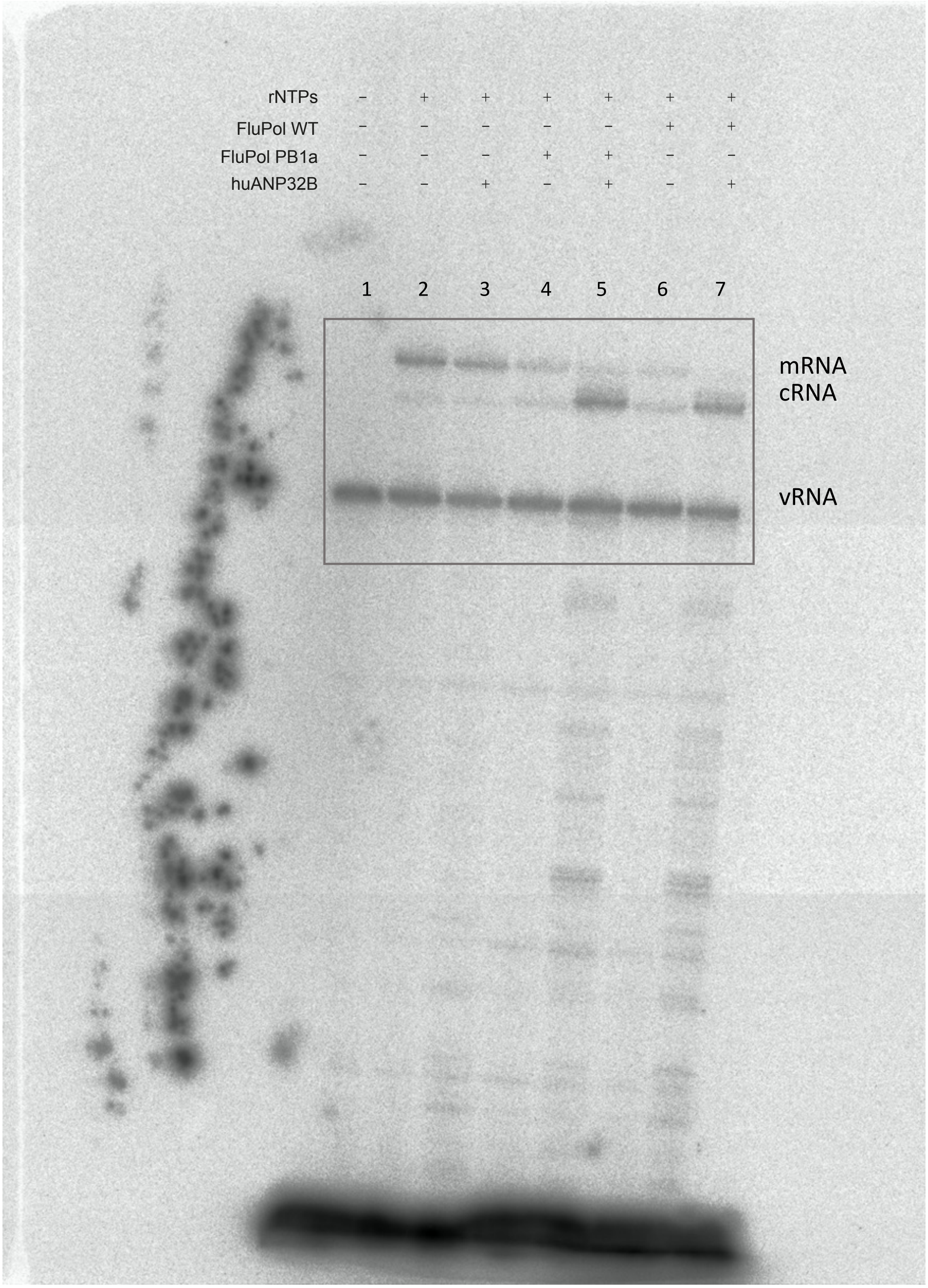

visualised by phosphorimaging on an FLA-5000 scanner

Fig 1B repeat 1

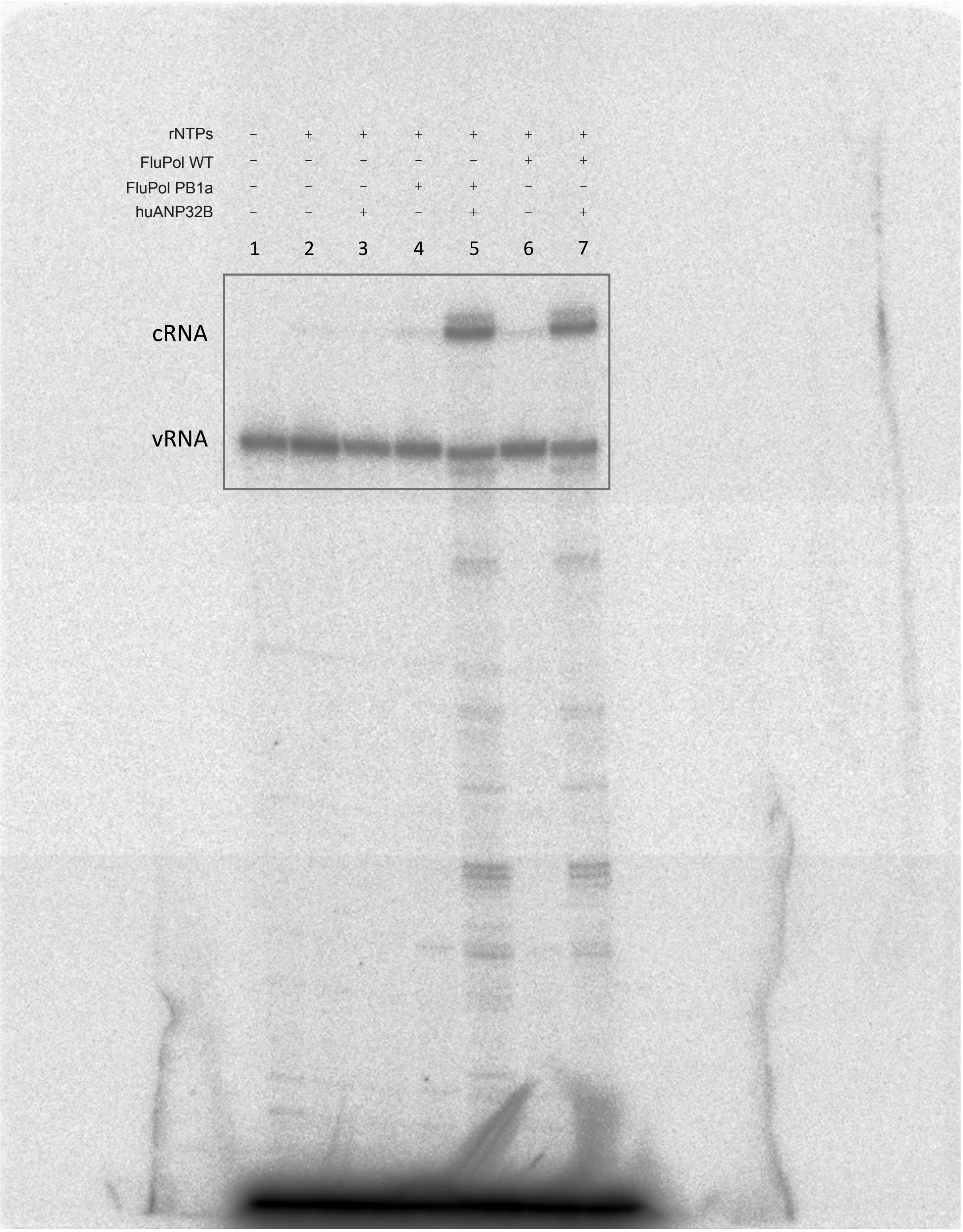

visualised by phosphorimaging on an FLA-5000 scanner

Fig 1B repeat 2

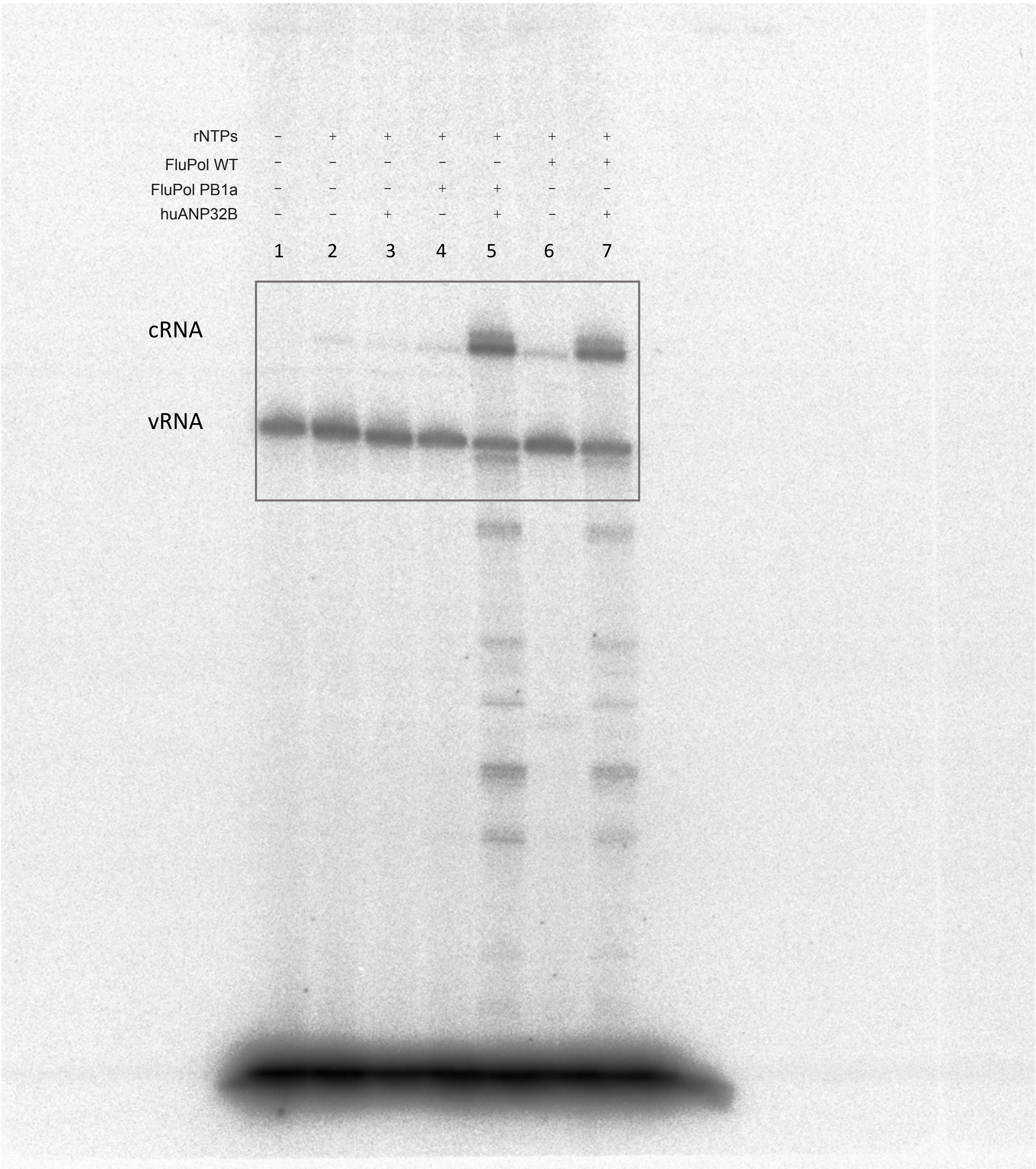

visualised by phosphorimaging on an FLA-5000 scanner

Fig 1B repeat 3

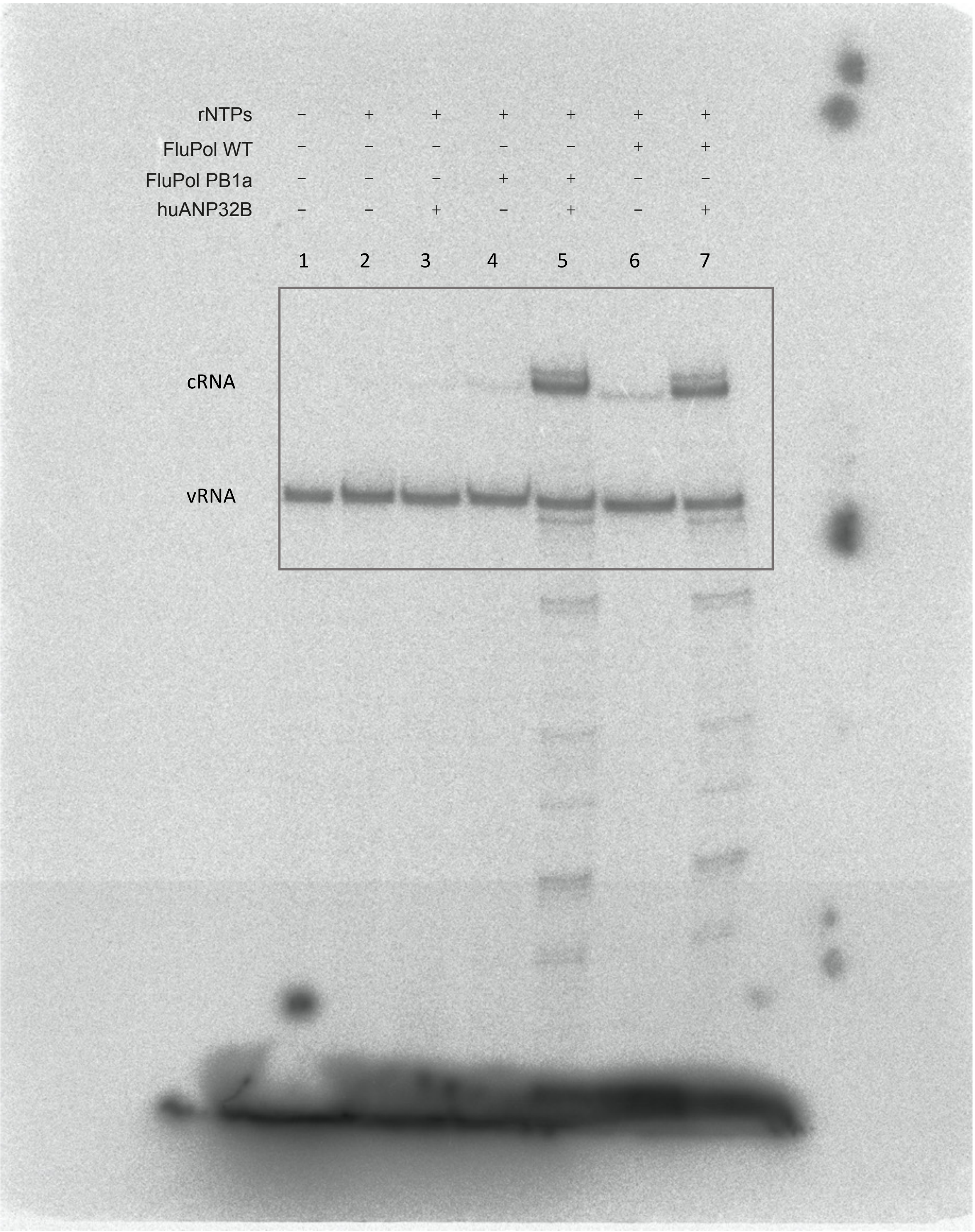

visualised by phosphorimaging on an FLA-5000 scanner

Fig 1C repeat 1

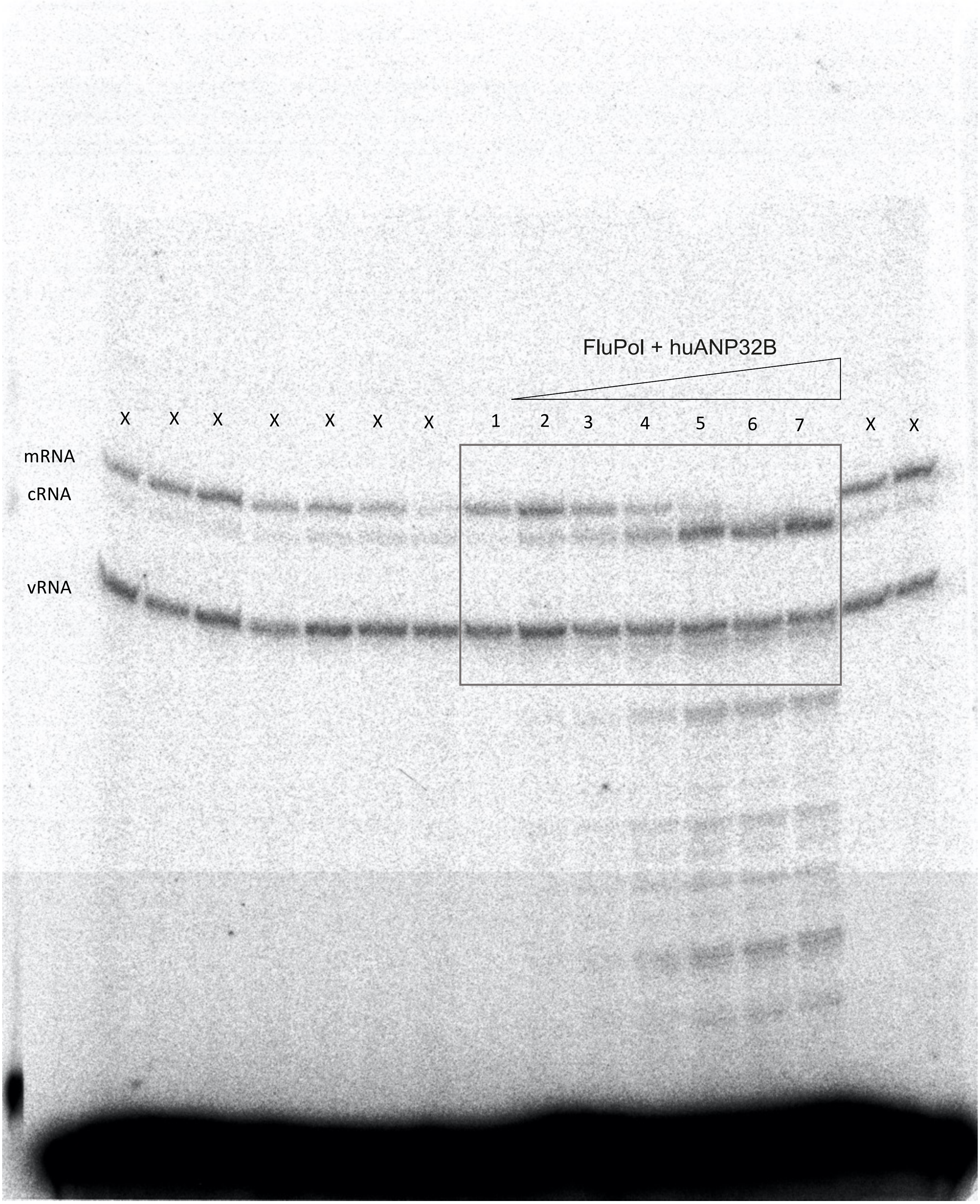

visualised by phosphorimaging on an FLA-5000 scanner

Fig 1C repeat 2

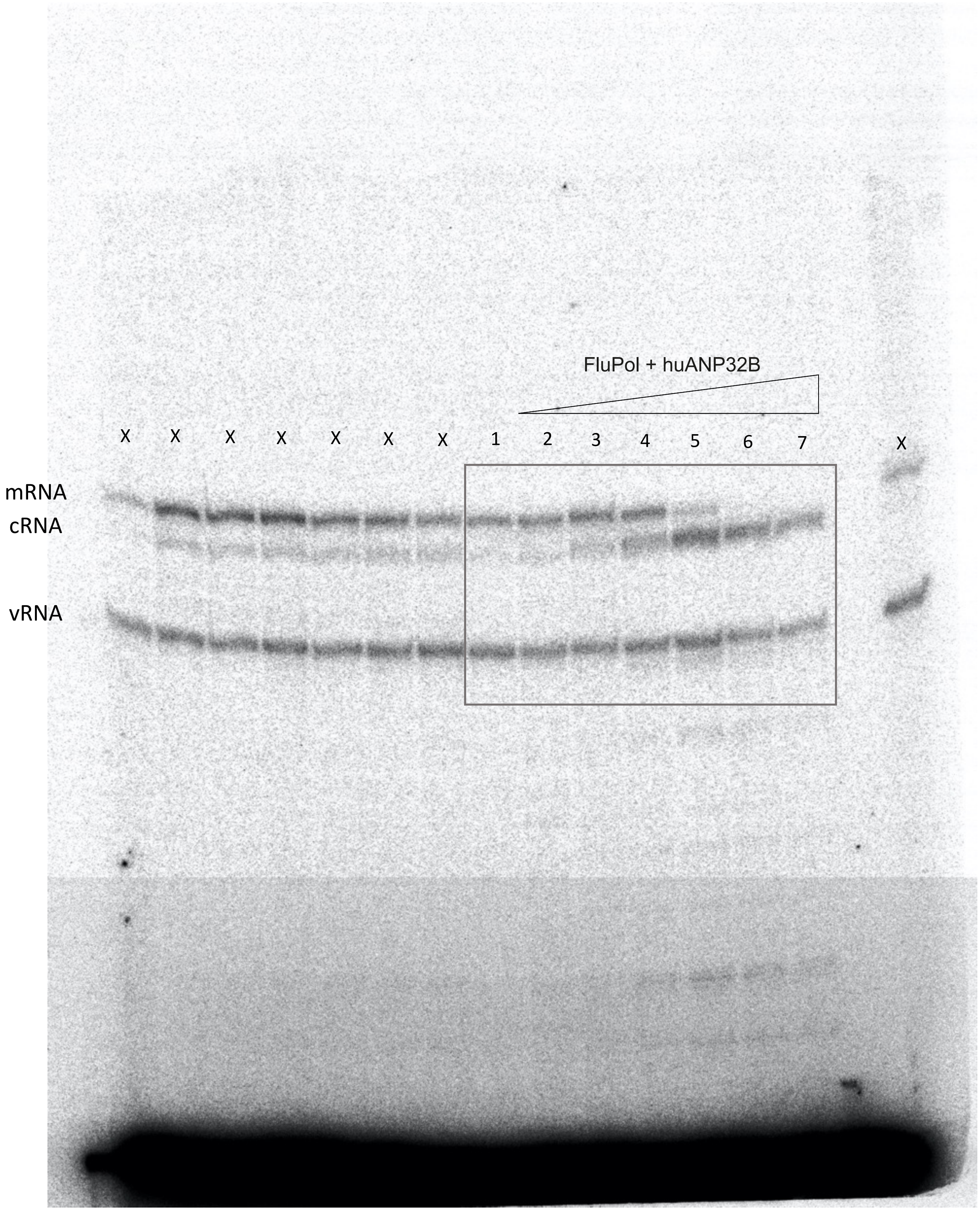

visualised by phosphorimaging on an FLA-5000 scanner

Fig 1C repeat 3

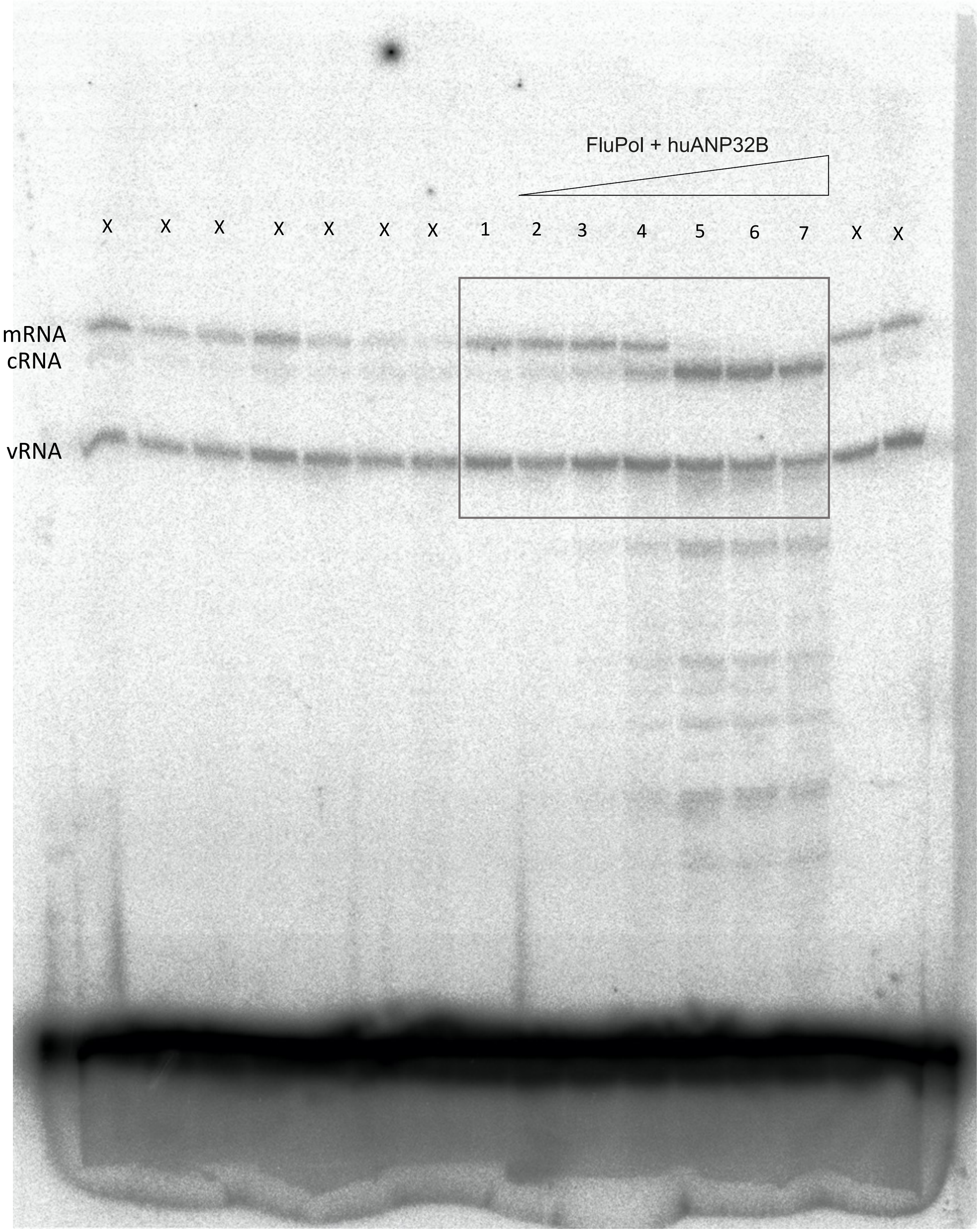

visualised by phosphorimaging on an FLA-5000 scanner

Fig 1D repeat 1

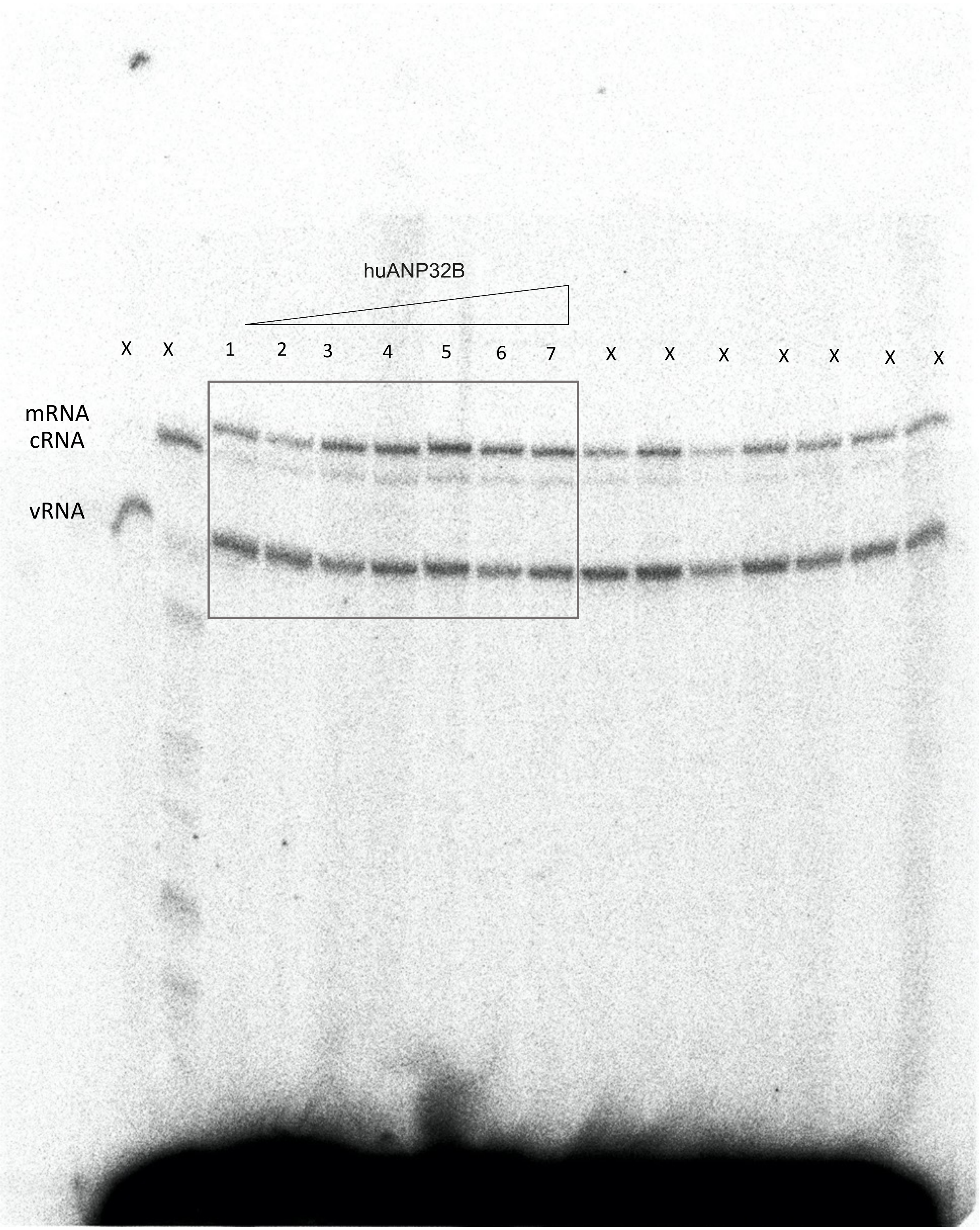

visualised by phosphorimaging on an FLA-5000 scanner

Fig 1D repeat 2

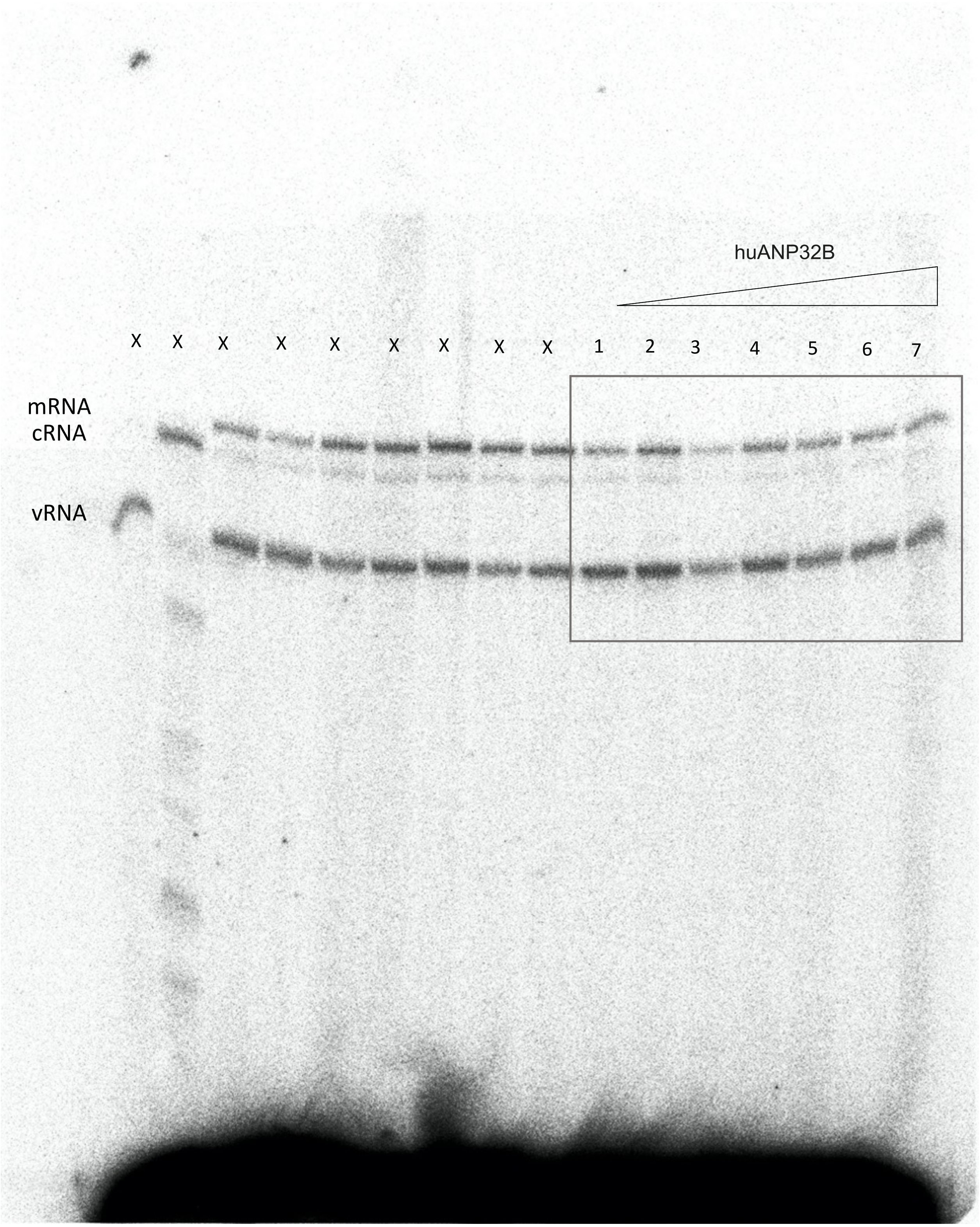

visualised by phosphorimaging on an FLA-5000 scanner

Fig 1D repeat 3

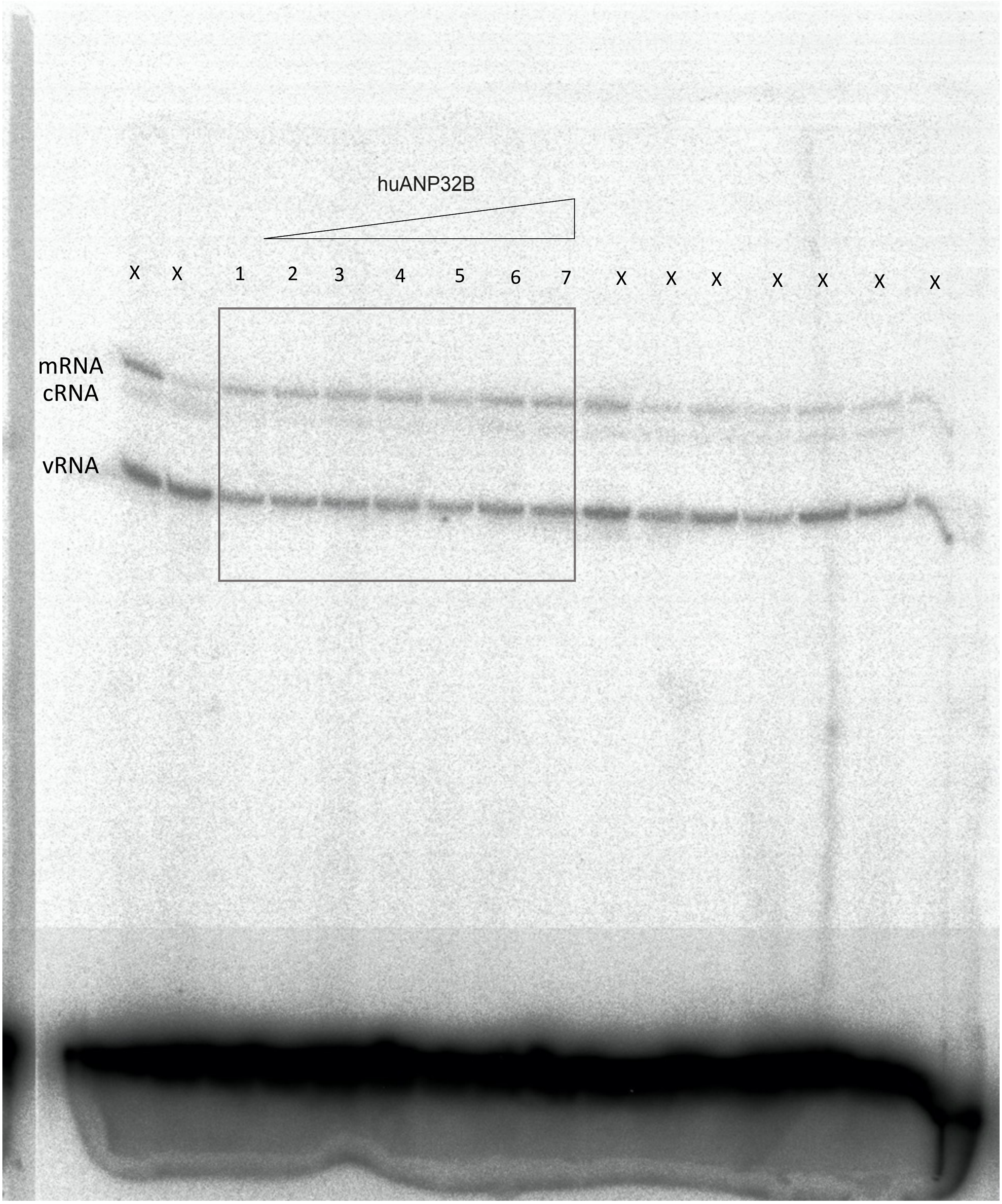

visualised by phosphorimaging on an FLA-5000 scanner

Fig 1E repeat 1

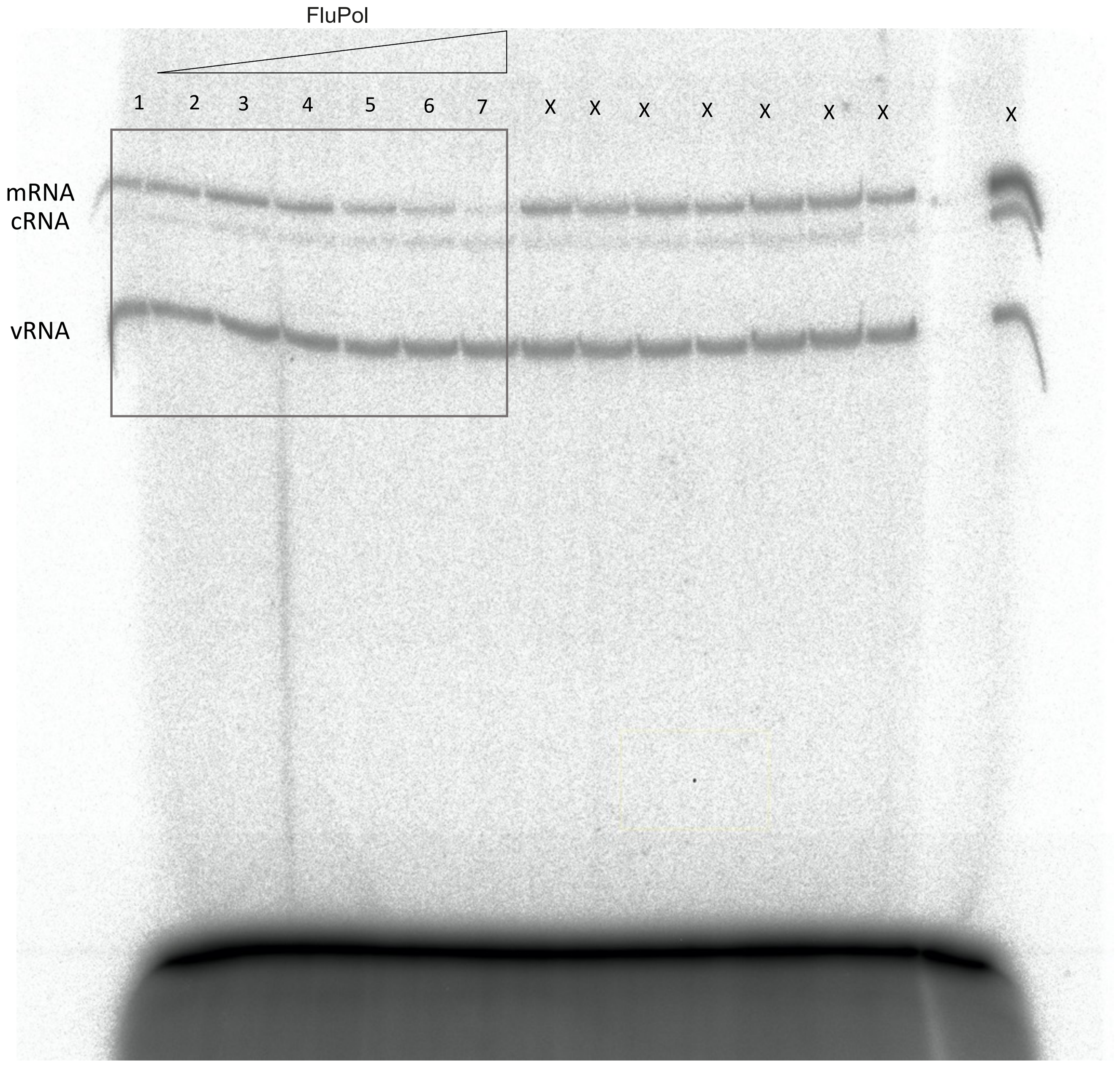

visualised by phosphorimaging on an FLA-5000 scanner

Fig 1E repeat 2

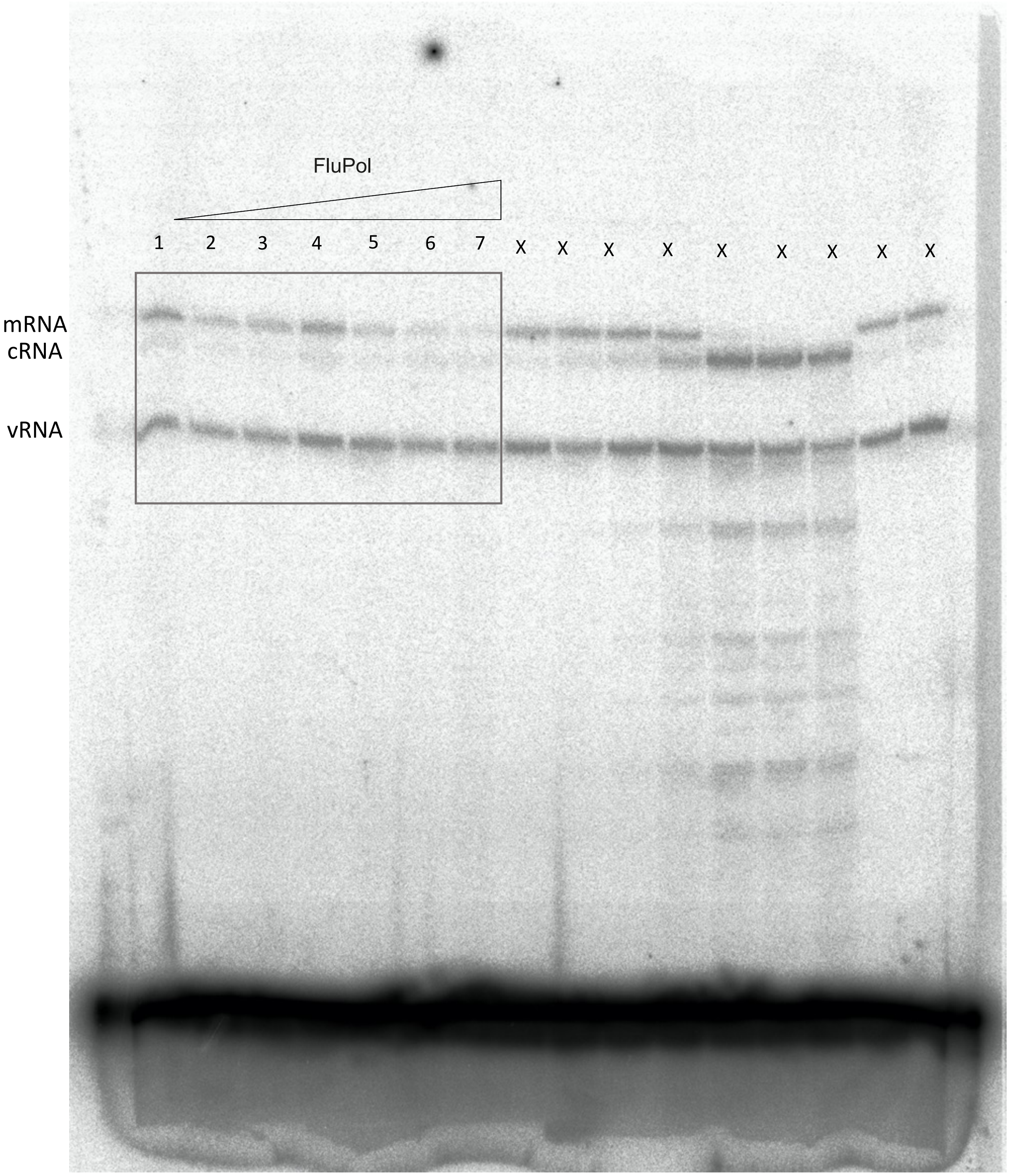

visualised by phosphorimaging on an FLA-5000 scanner

Fig 1E repeat 3

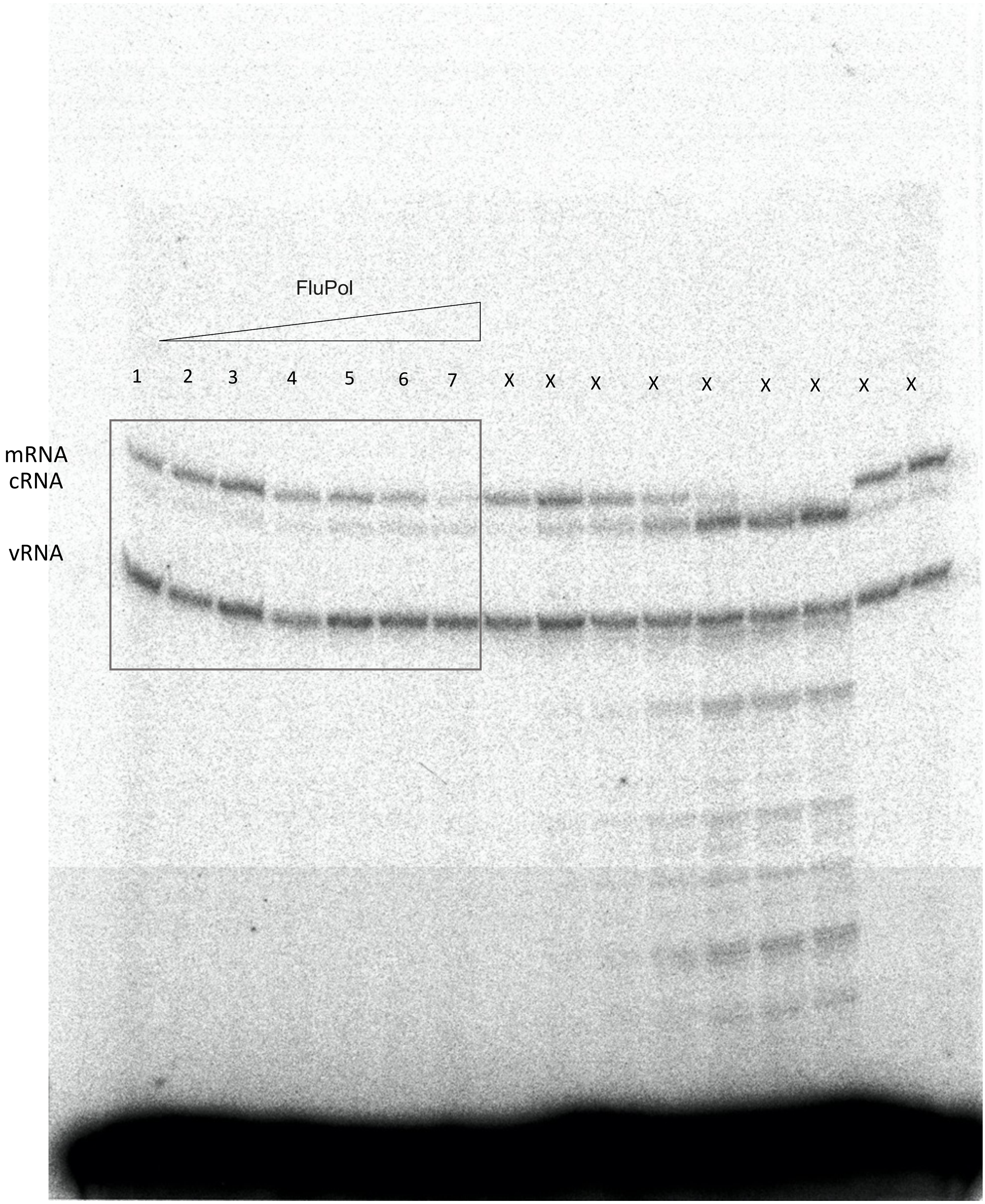

visualised by phosphorimaging on an FLA-5000 scanner

Fig 2A repeat 1

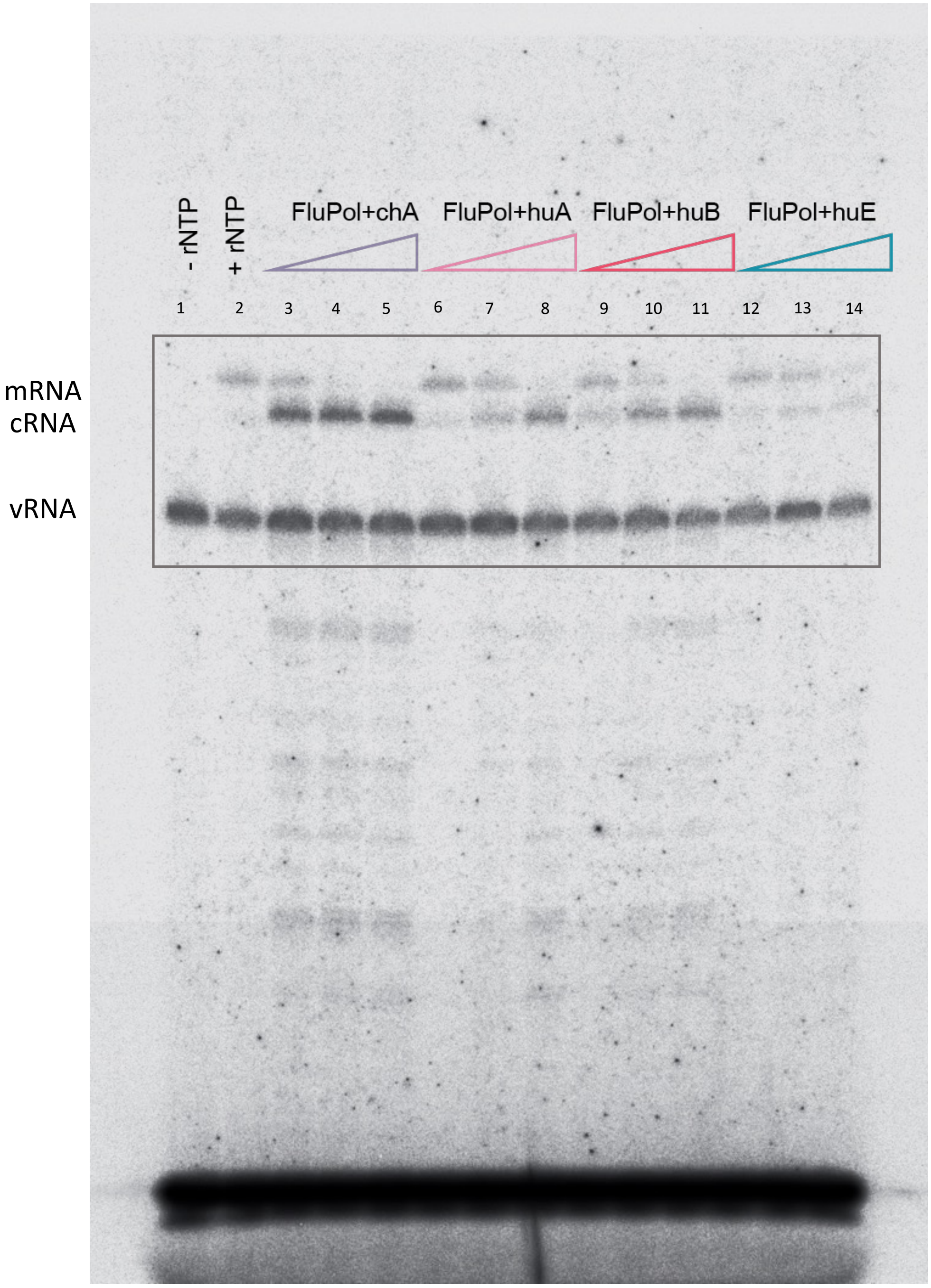

visualised by phosphorimaging on an FLA-5000 scanner

Fig 2A repeat 2

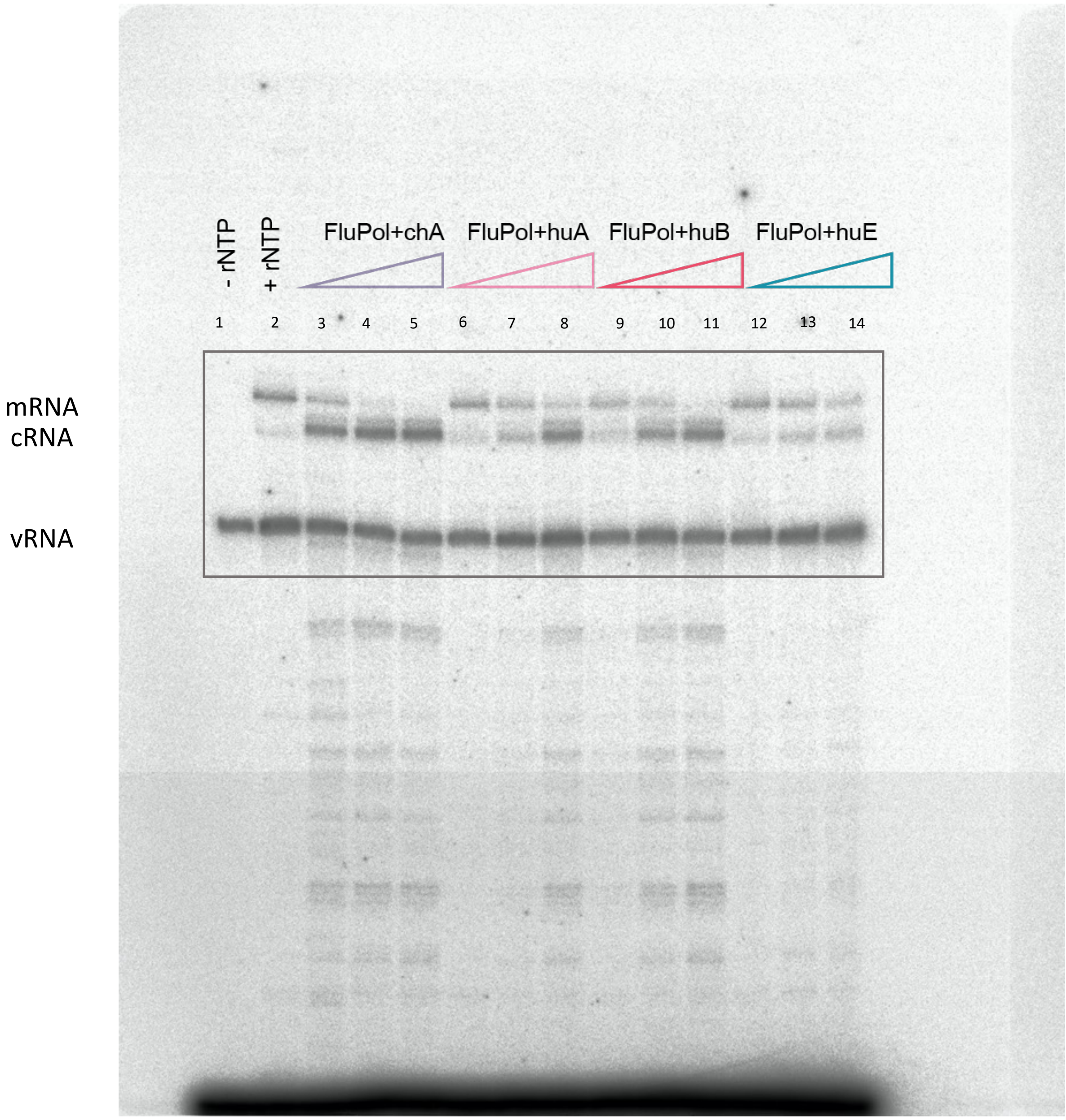

visualised by phosphorimaging on an FLA-5000 scanner

Fig 2A repeat 3

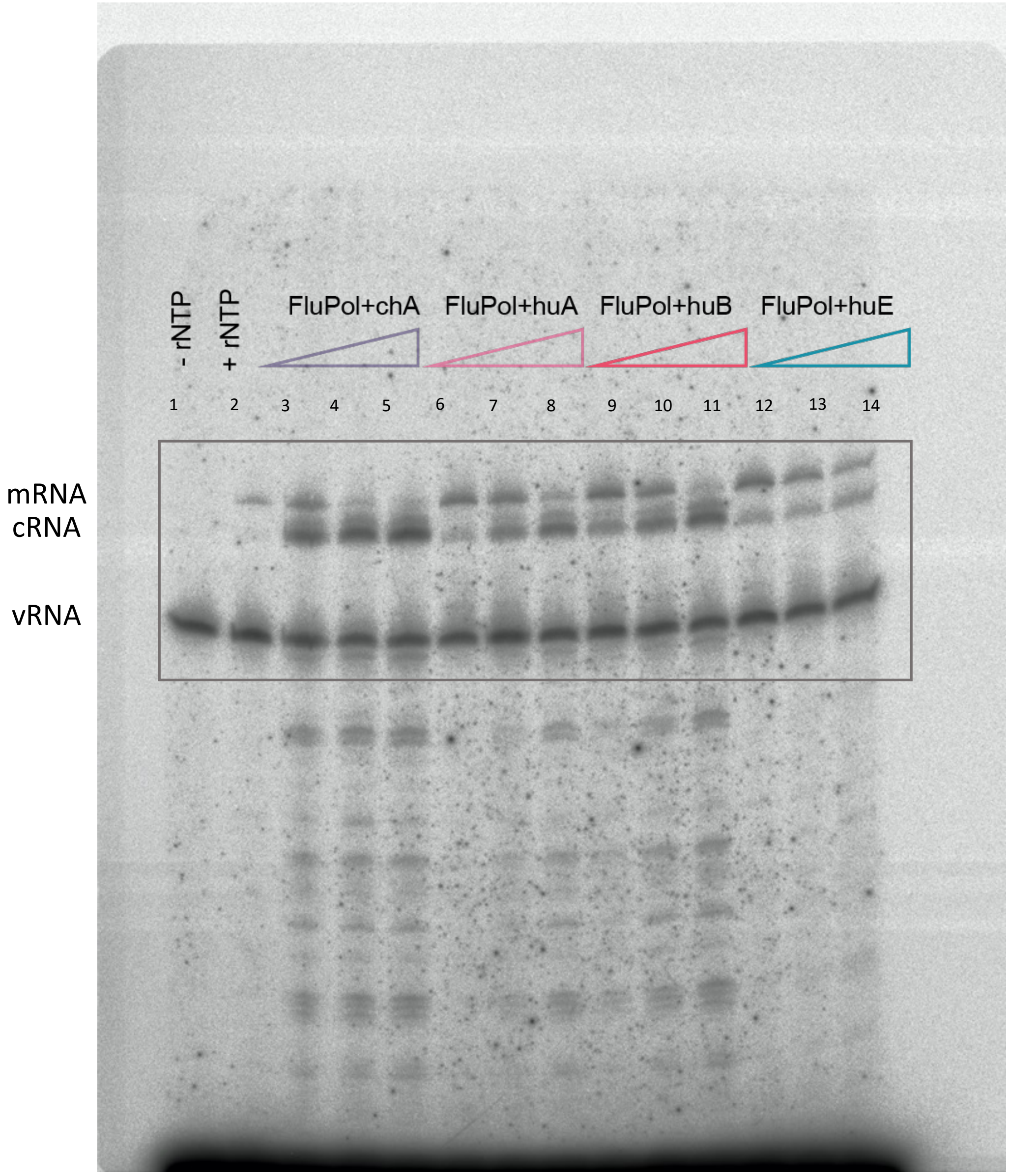

visualised by phosphorimaging on an FLA-5000 scanner

Fig 2B repeat 1

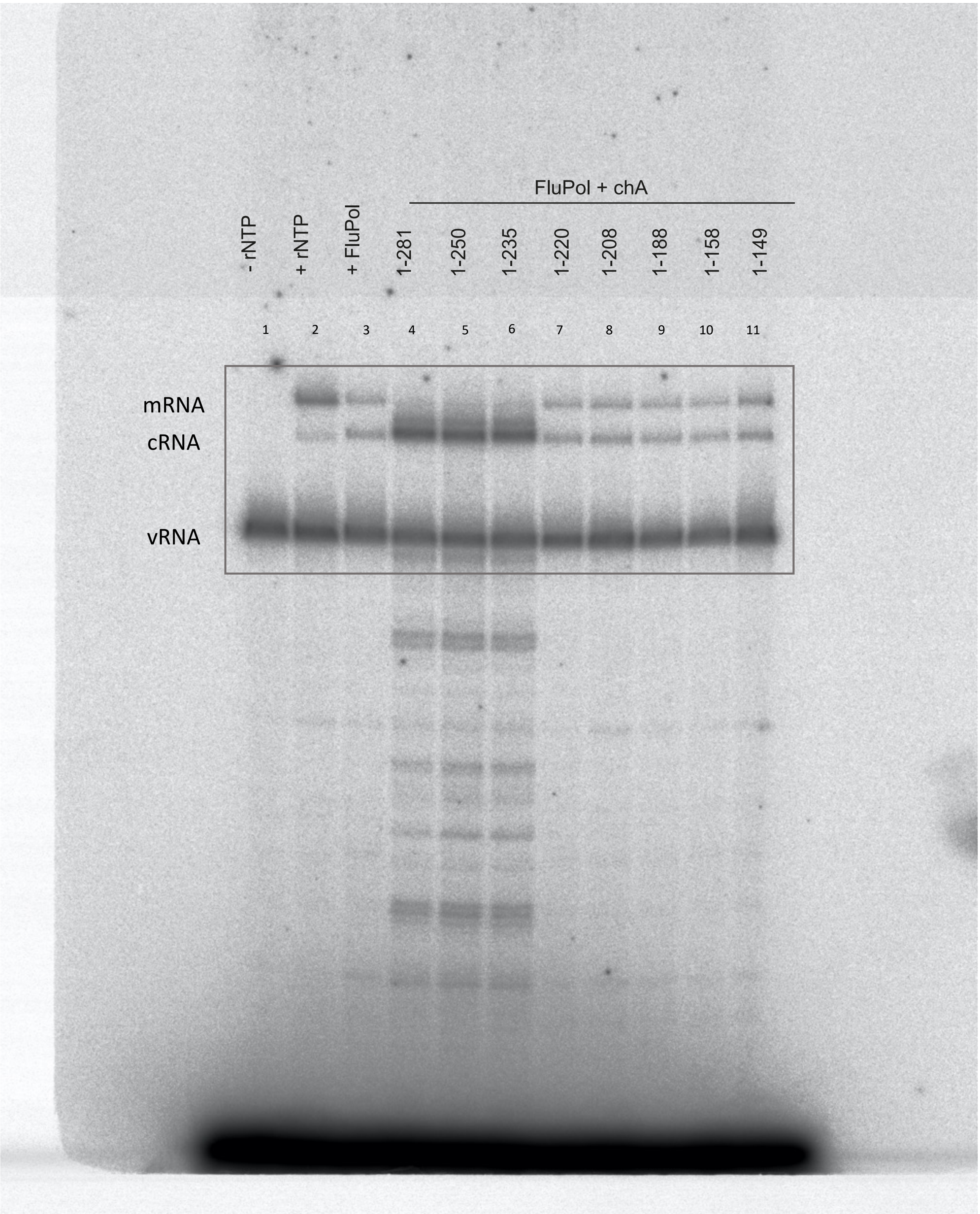

visualised by phosphorimaging on an FLA-5000 scanner

Fig 2B repeat 2

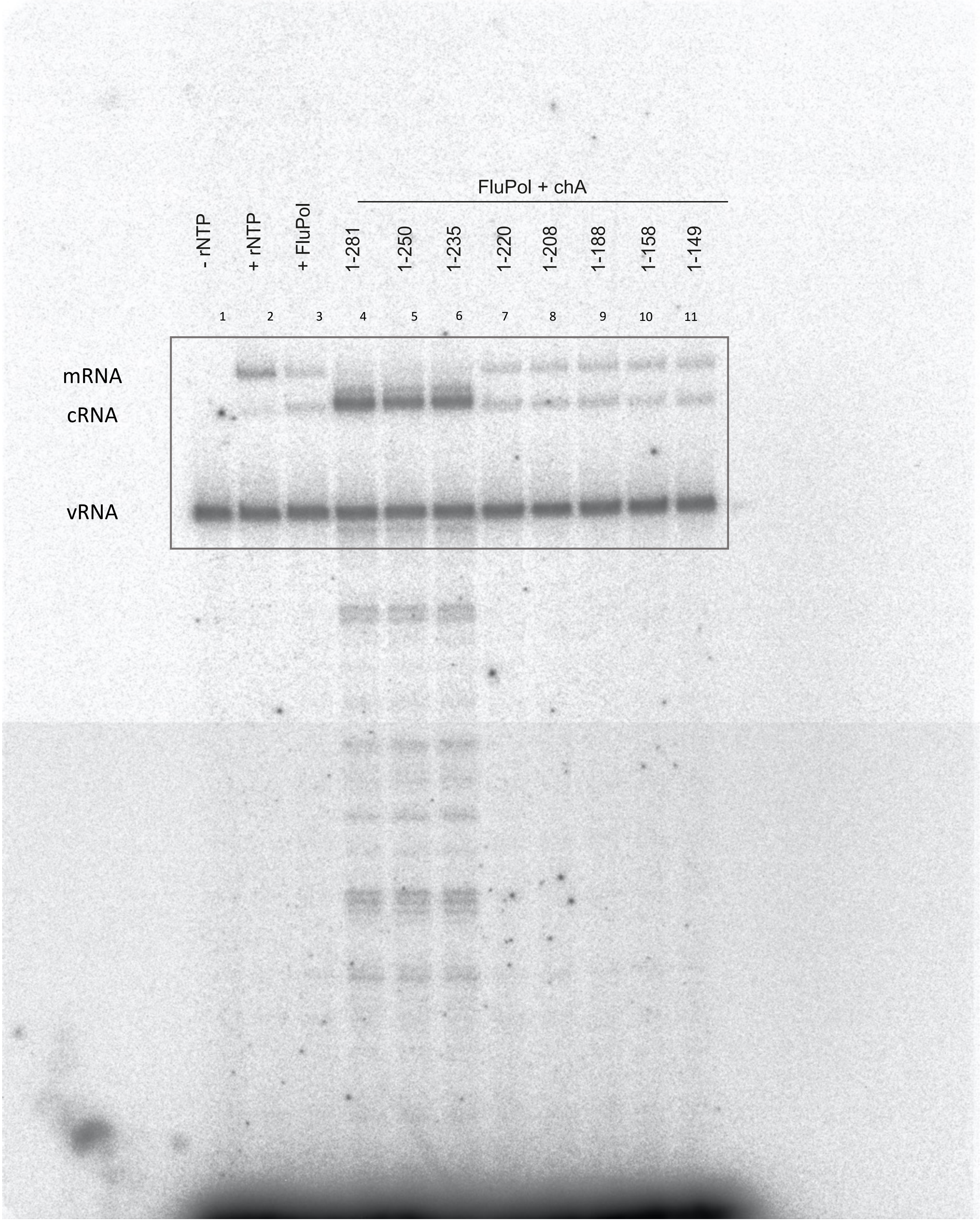

visualised by phosphorimaging on an FLA-5000 scanner

Fig 2B repeat 3

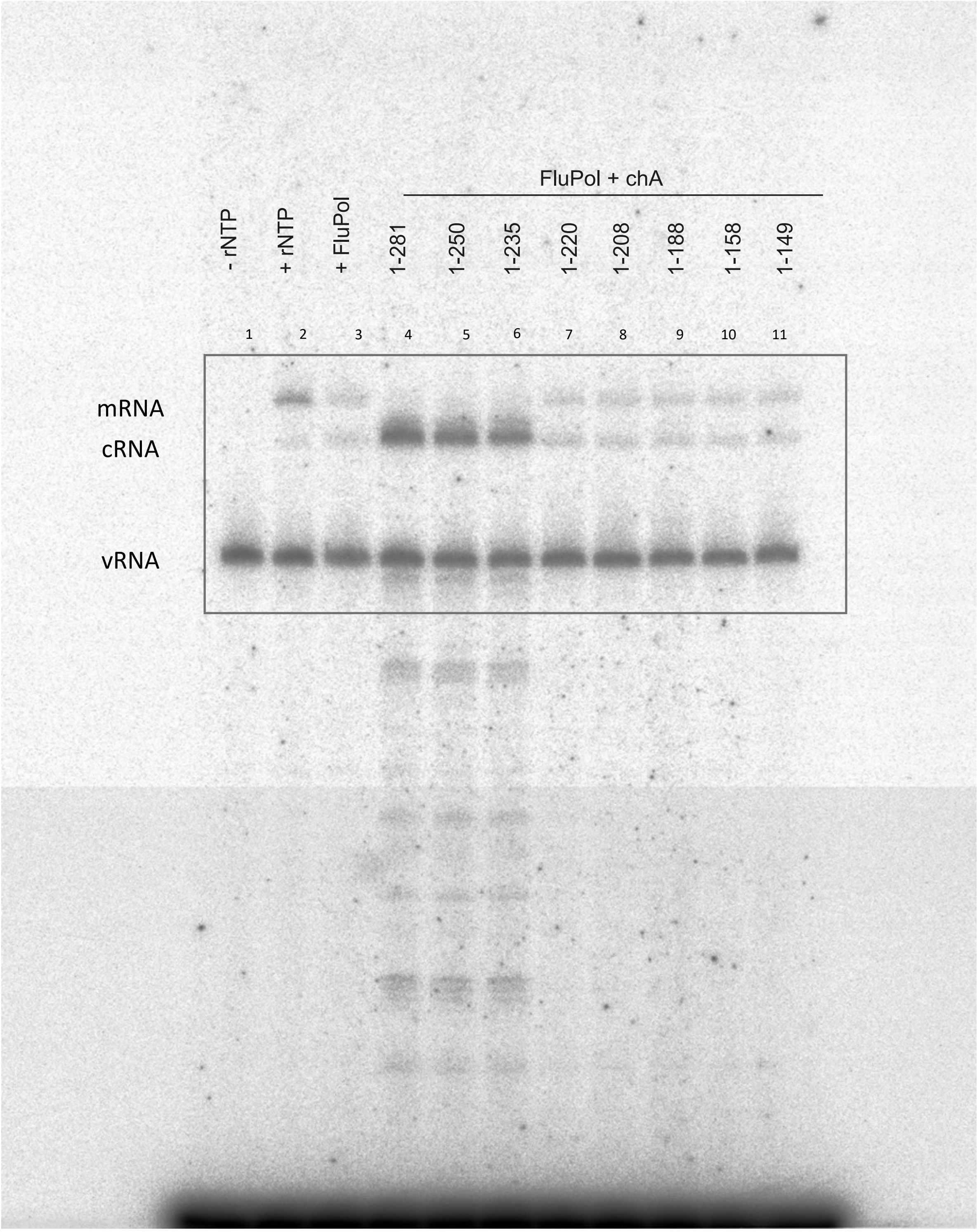

visualised by phosphorimaging on an FLA-5000 scanner

Fig 3A repeat 1

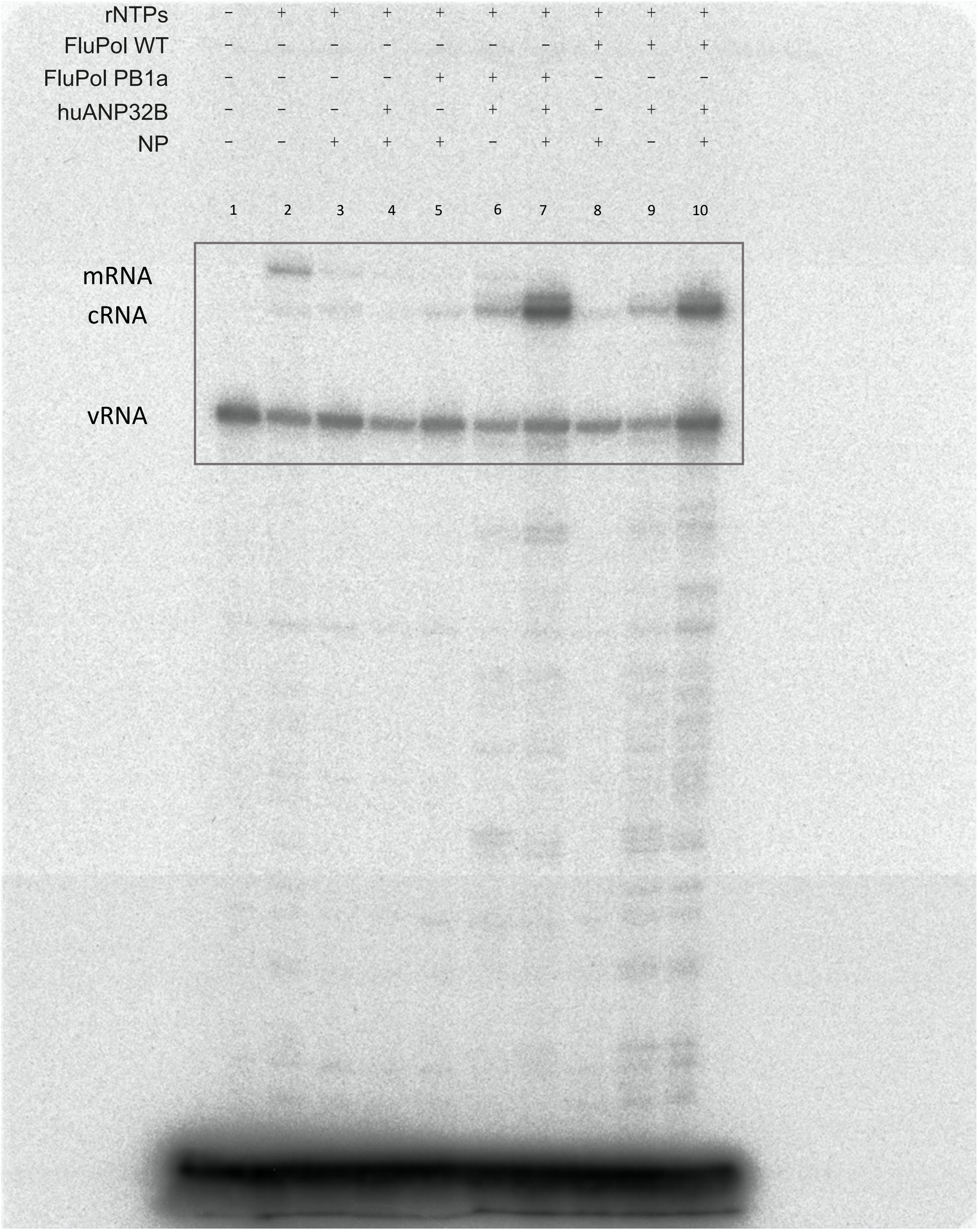

visualised by phosphorimaging on an FLA-5000 scanner

Fig 3A repeat 2

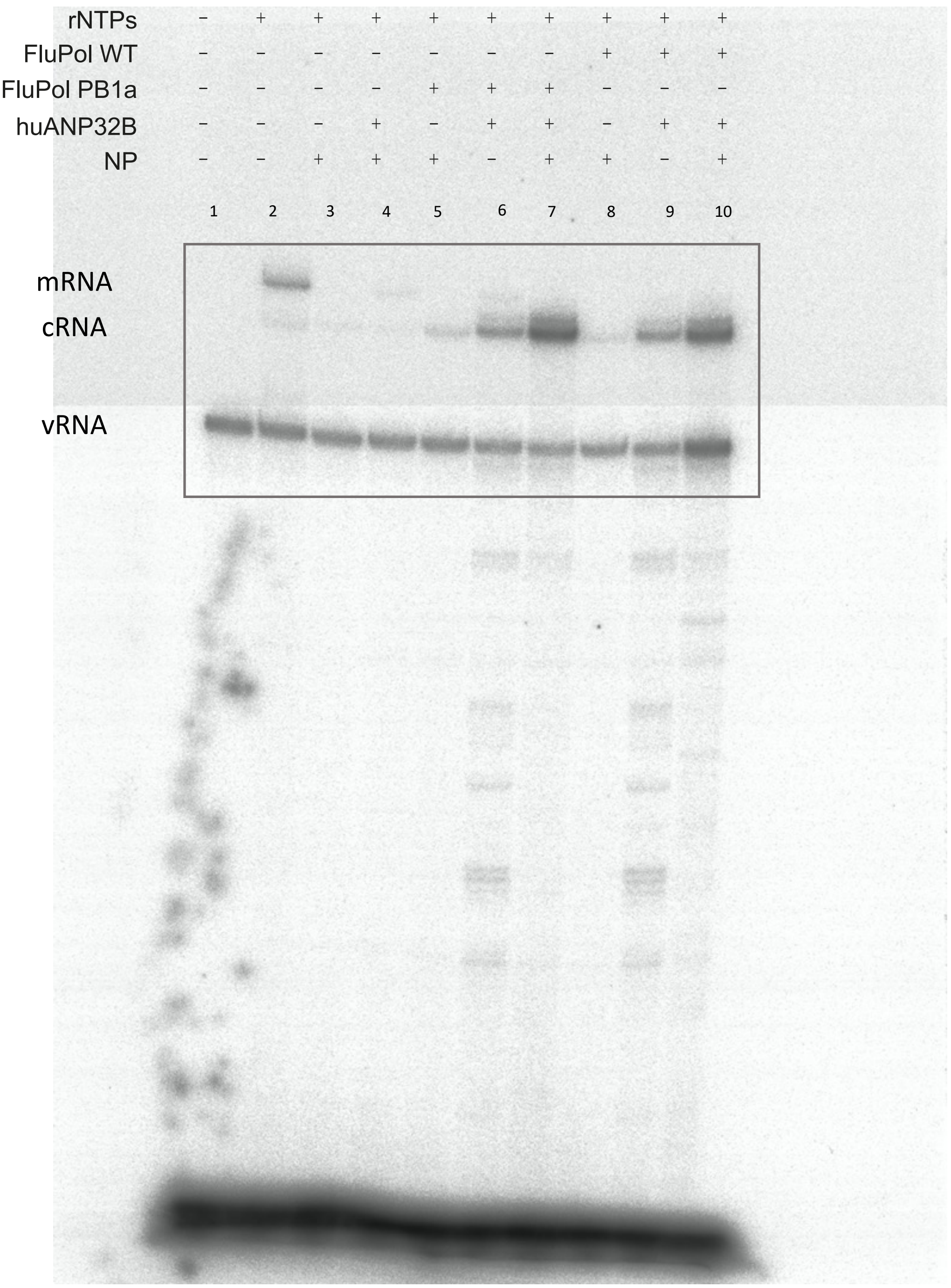

visualised by phosphorimaging on an FLA-5000 scanner

Fig 3A repeat 3

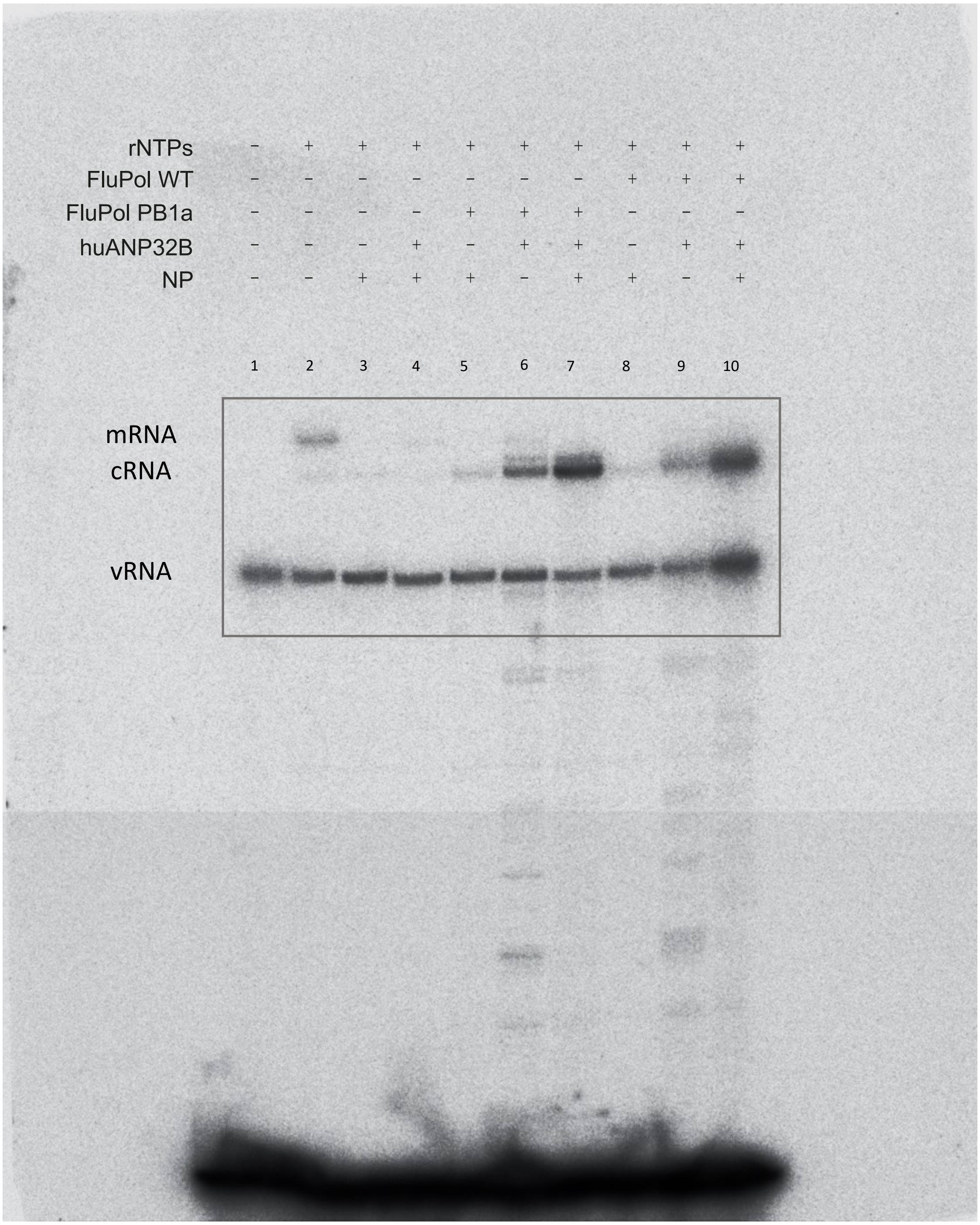

visualised by phosphorimaging on an FLA-5000 scanner

Fig 3B repeat 1

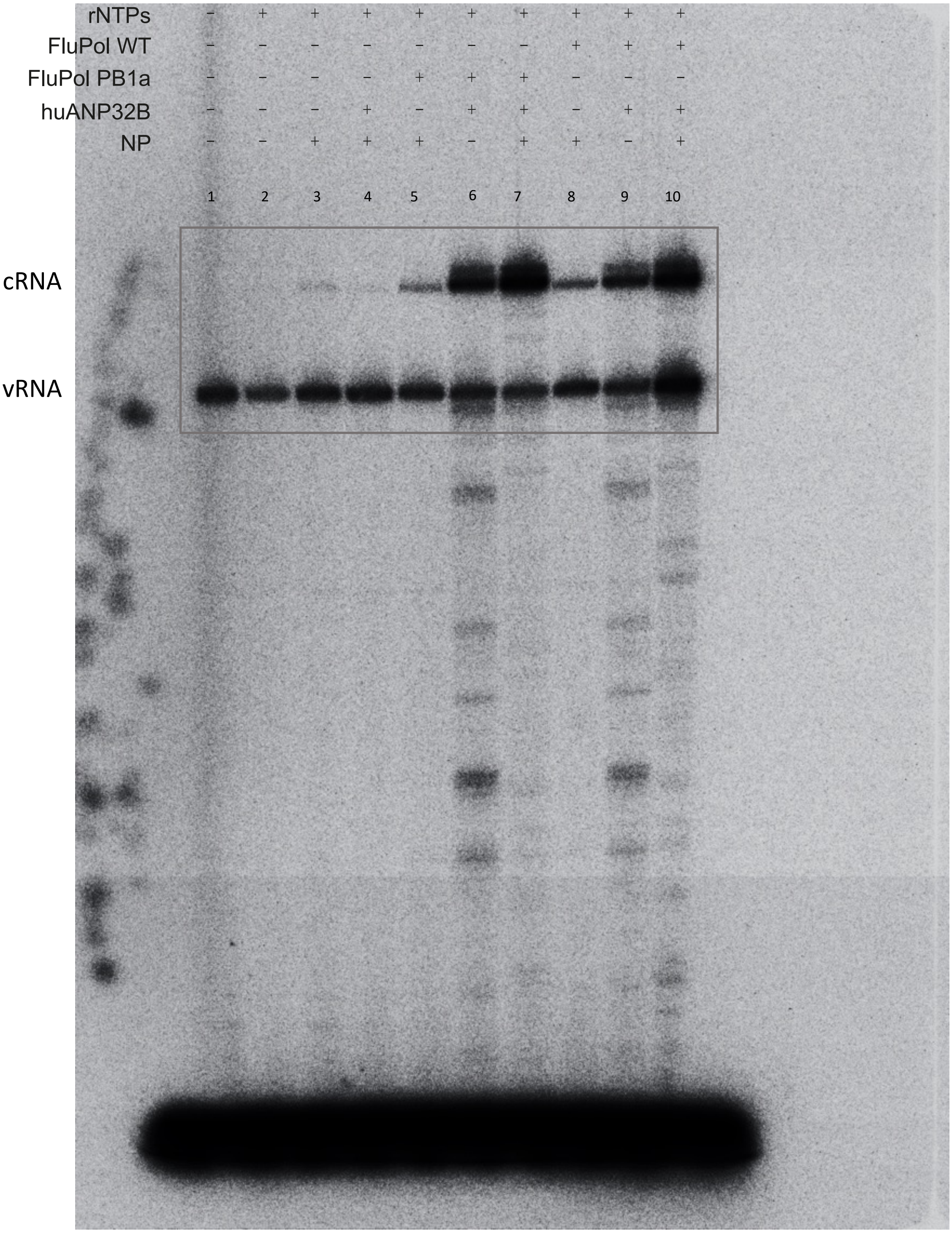

visualised by phosphorimaging on an FLA-5000 scanner

Fig 3B repeat 2

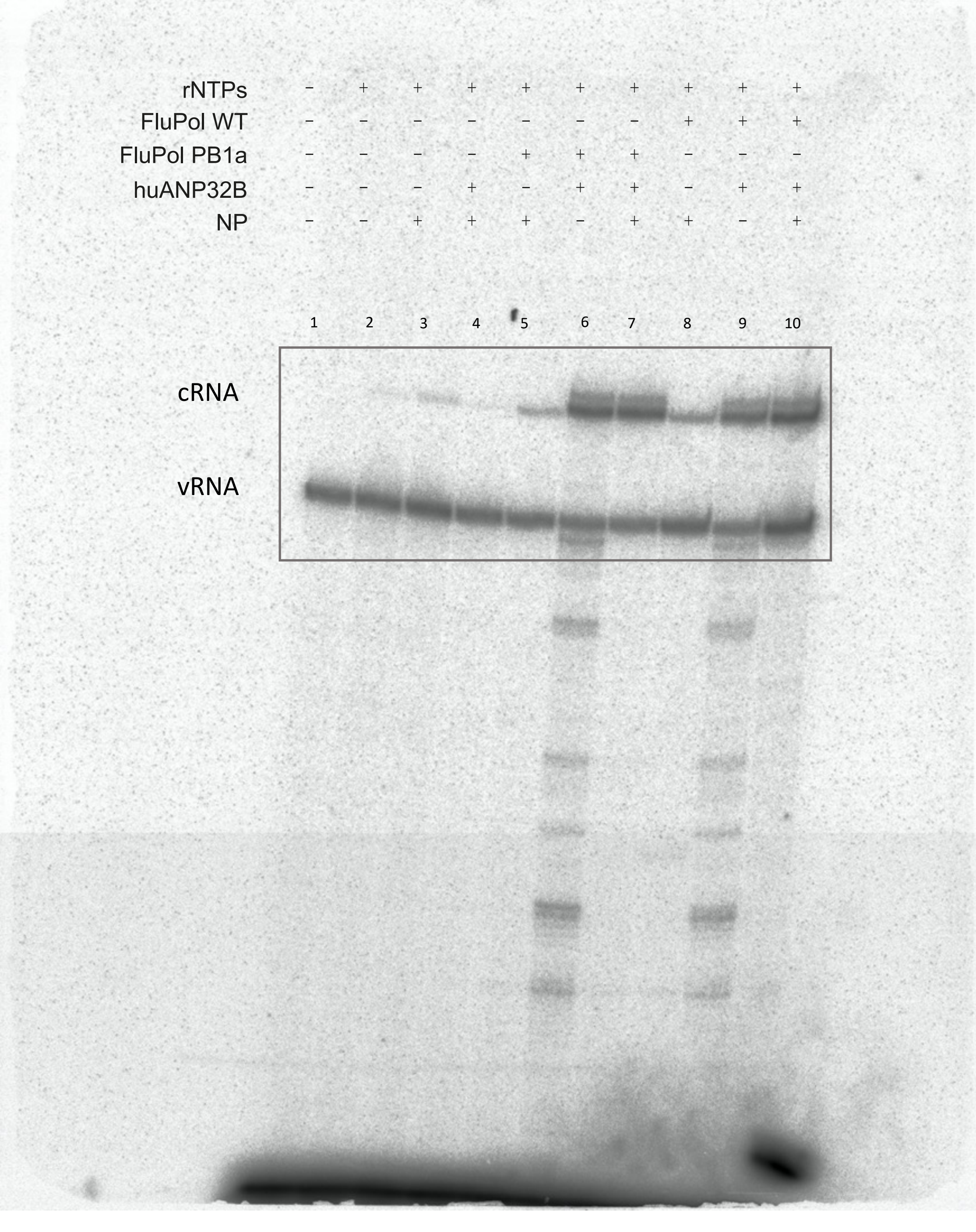

visualised by phosphorimaging on an FLA-5000 scanner

Fig 3B repeat 3

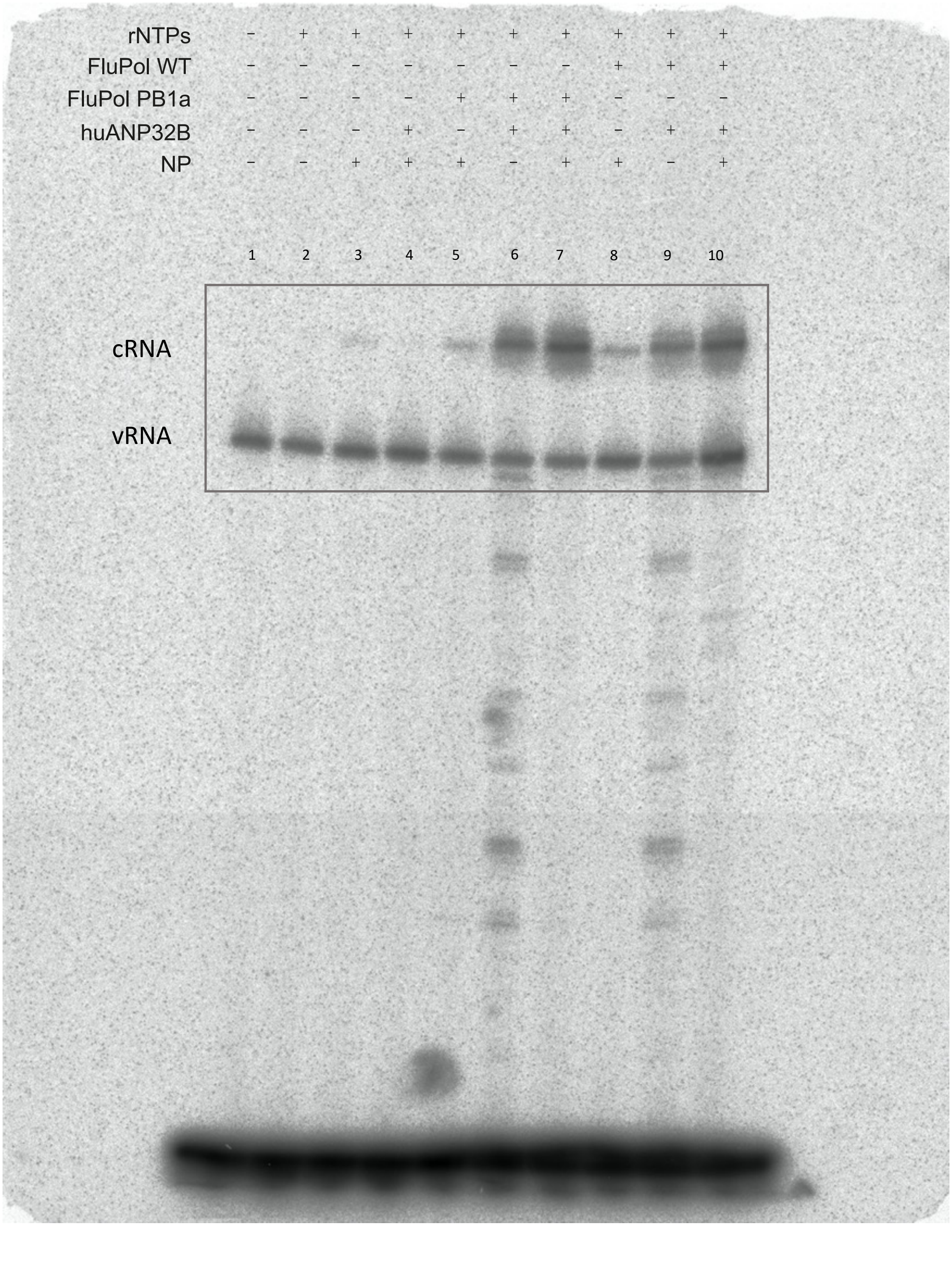

visualised by phosphorimaging on an FLA-5000 scanner

Fig 3C repeat 1

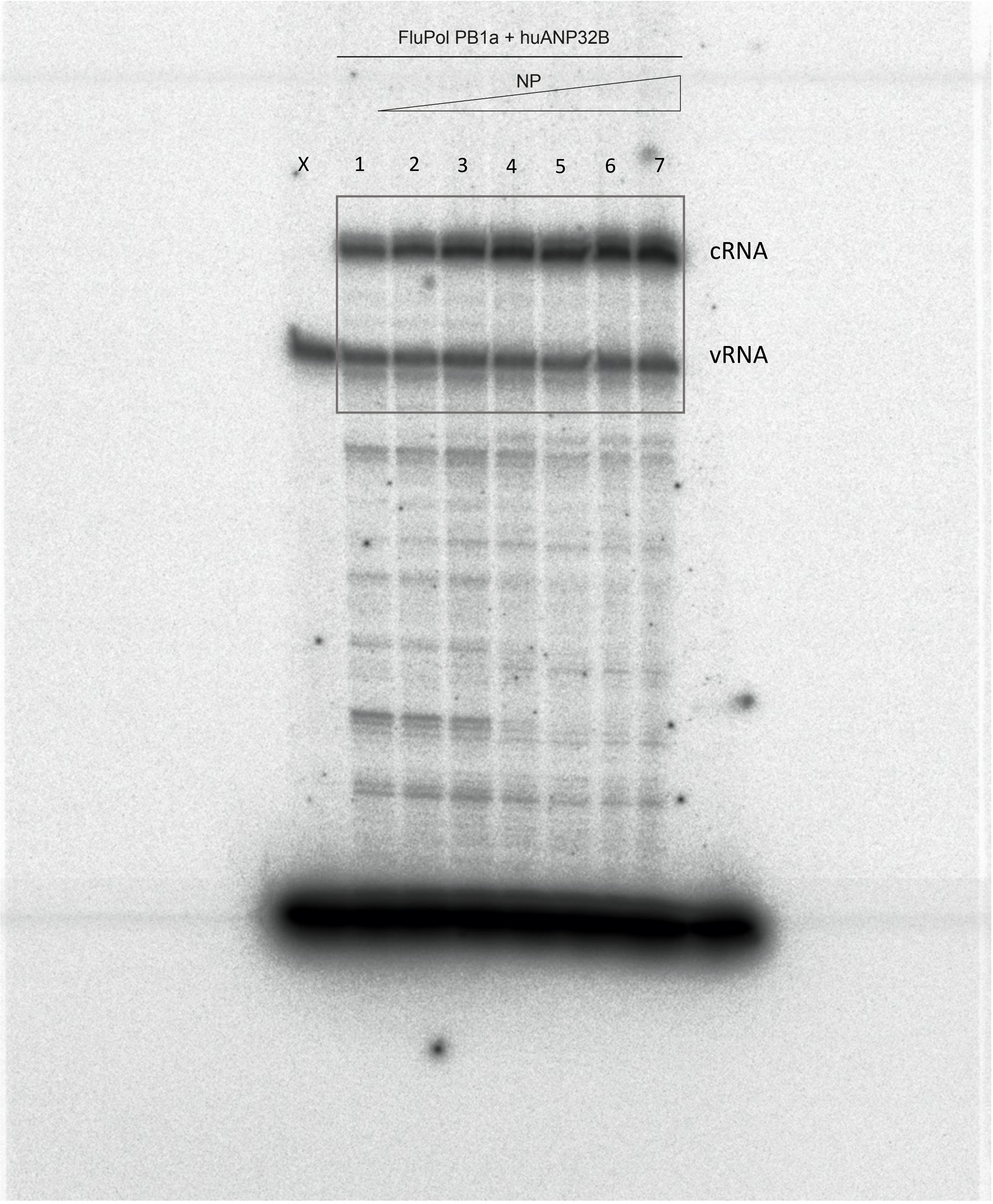

visualised by phosphorimaging on an FLA-5000 scanner

Fig 3C repeat 2

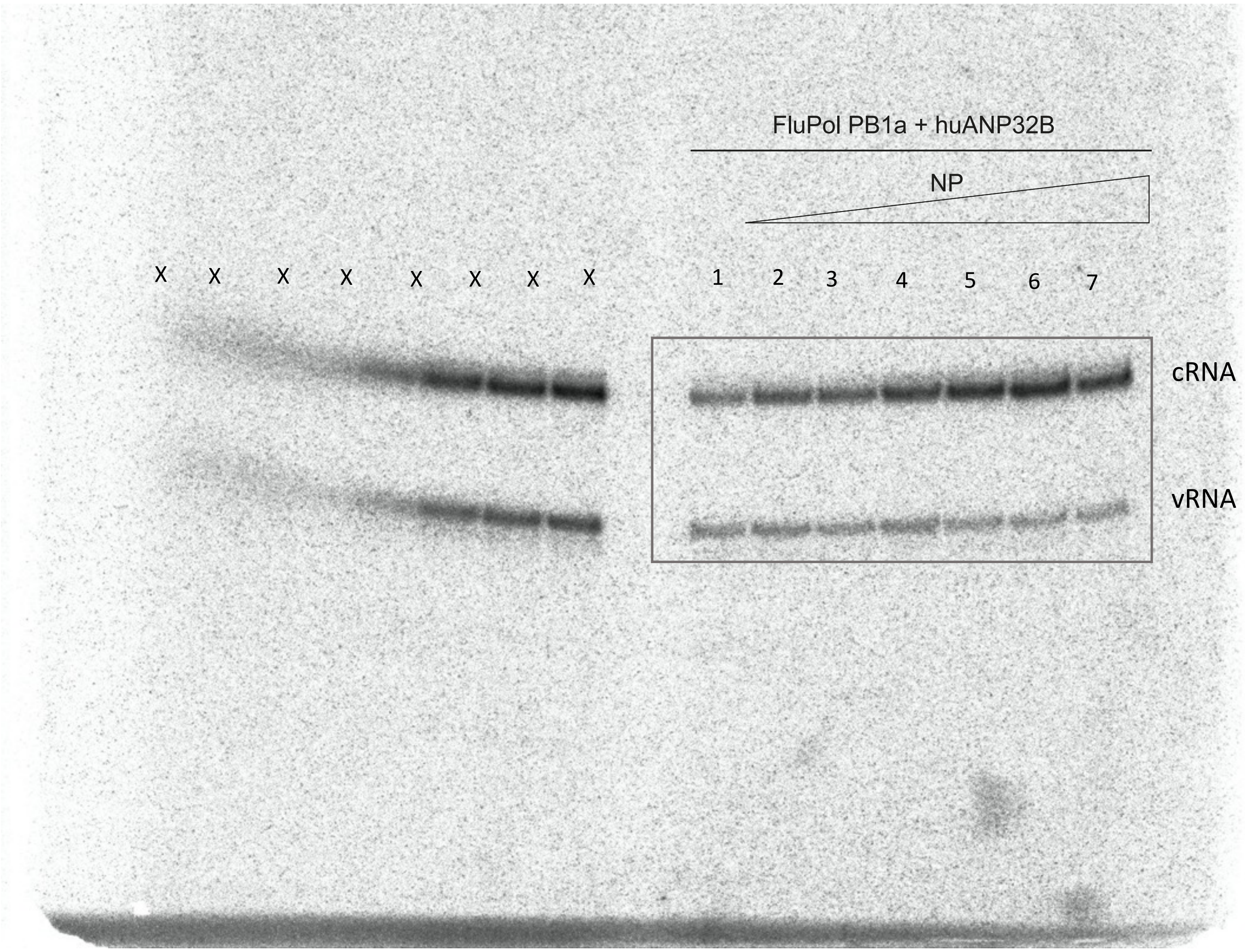

visualised by phosphorimaging on an FLA-5000 scanner

Fig 3C repeat 3

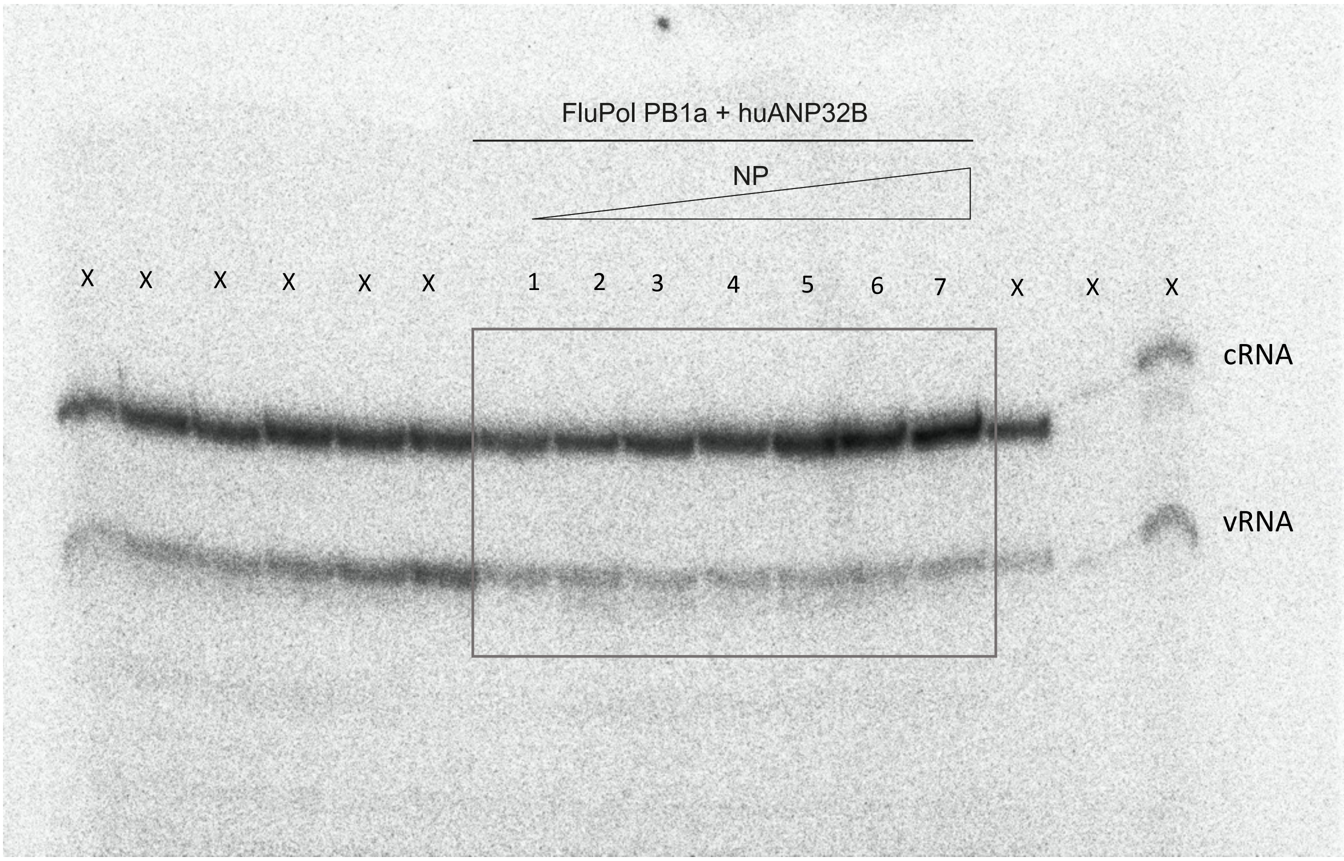

visualised by phosphorimaging on an FLA-5000 scanner

Fig 3D repeat 1

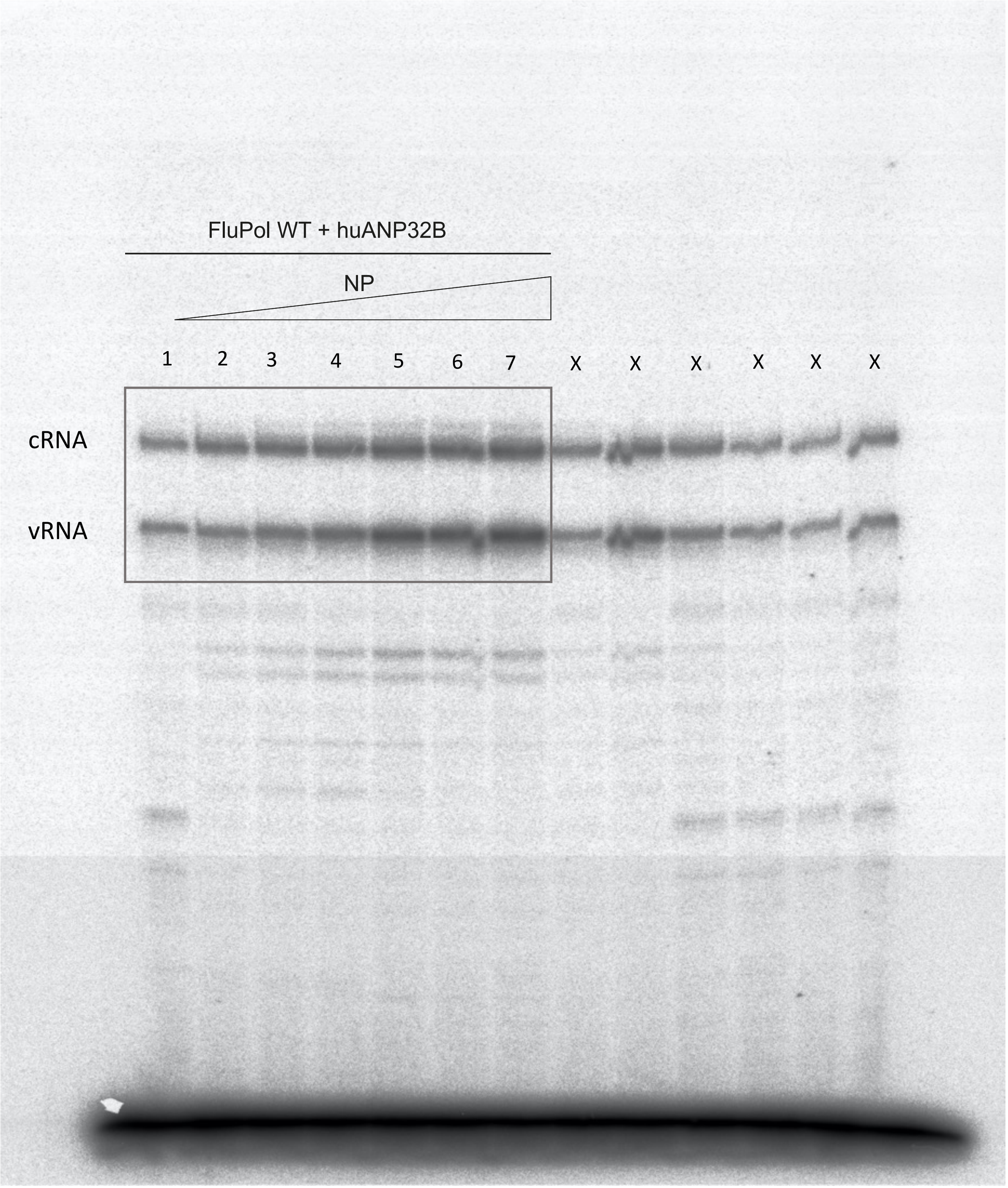

visualised by phosphorimaging on an FLA-5000 scanner

Fig 3D repeat 2

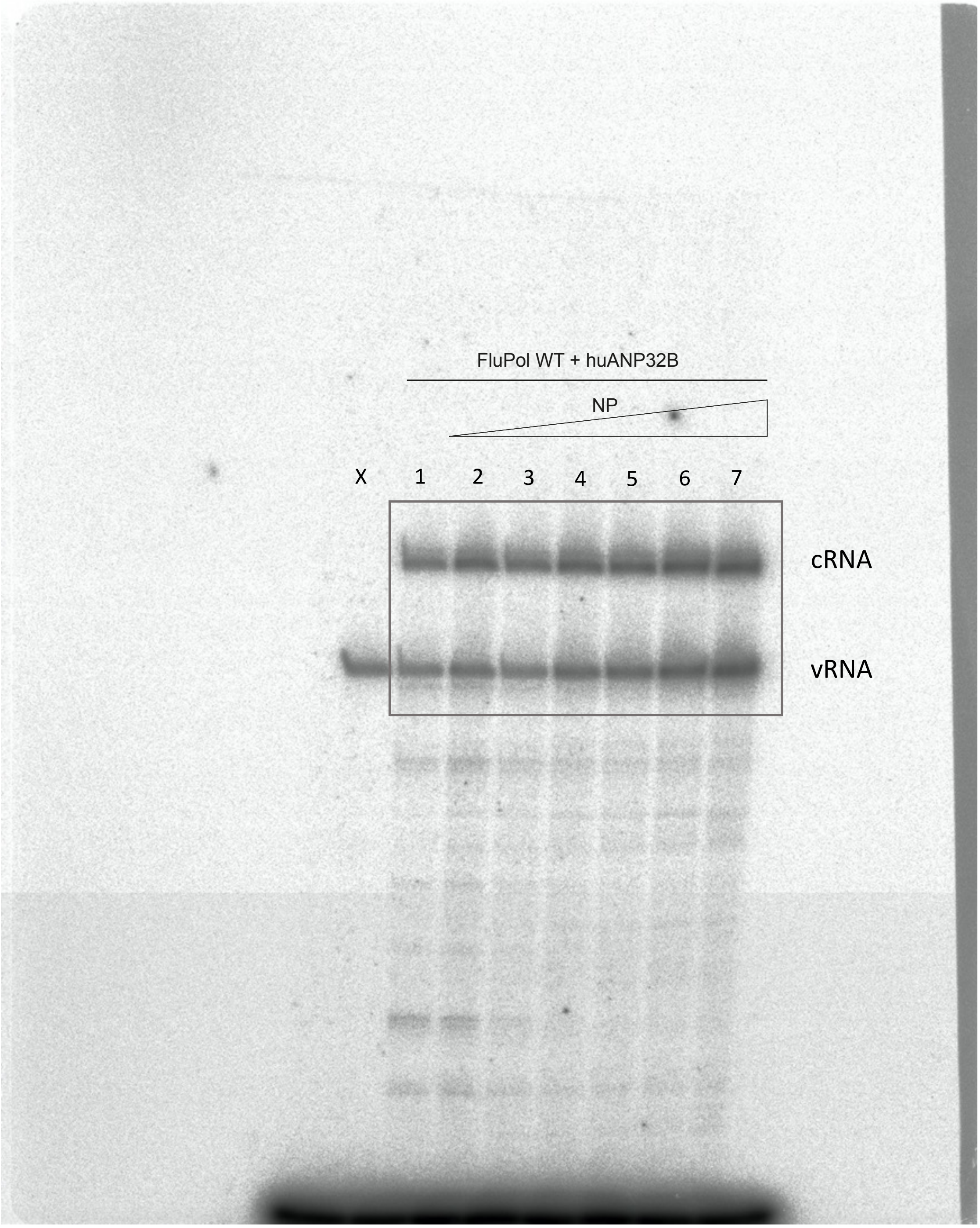

visualised by phosphorimaging on an FLA-5000 scanner

Fig 3D repeat 3

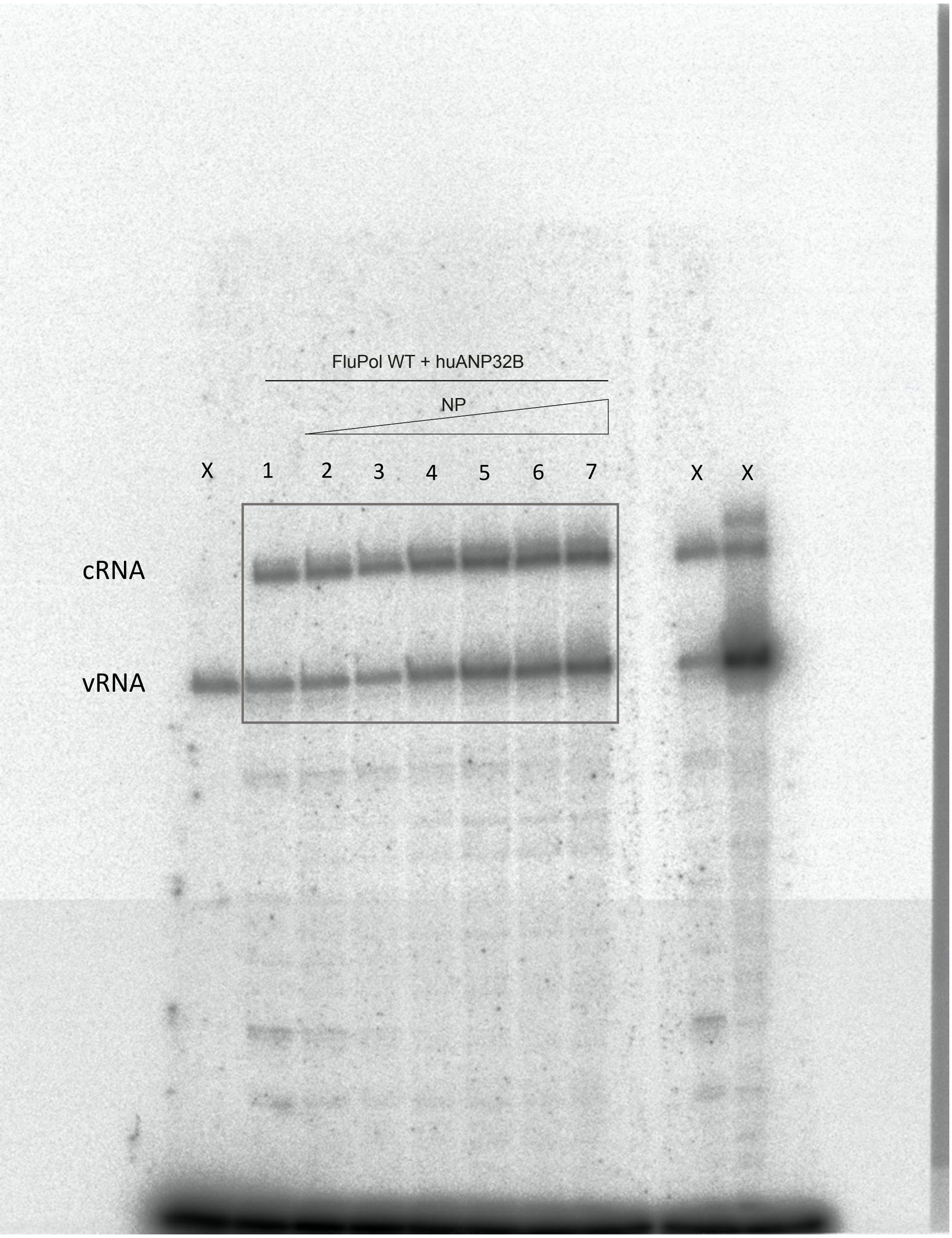

visualised by phosphorimaging on an FLA-5000 scanner

Fig 3E repeat 1

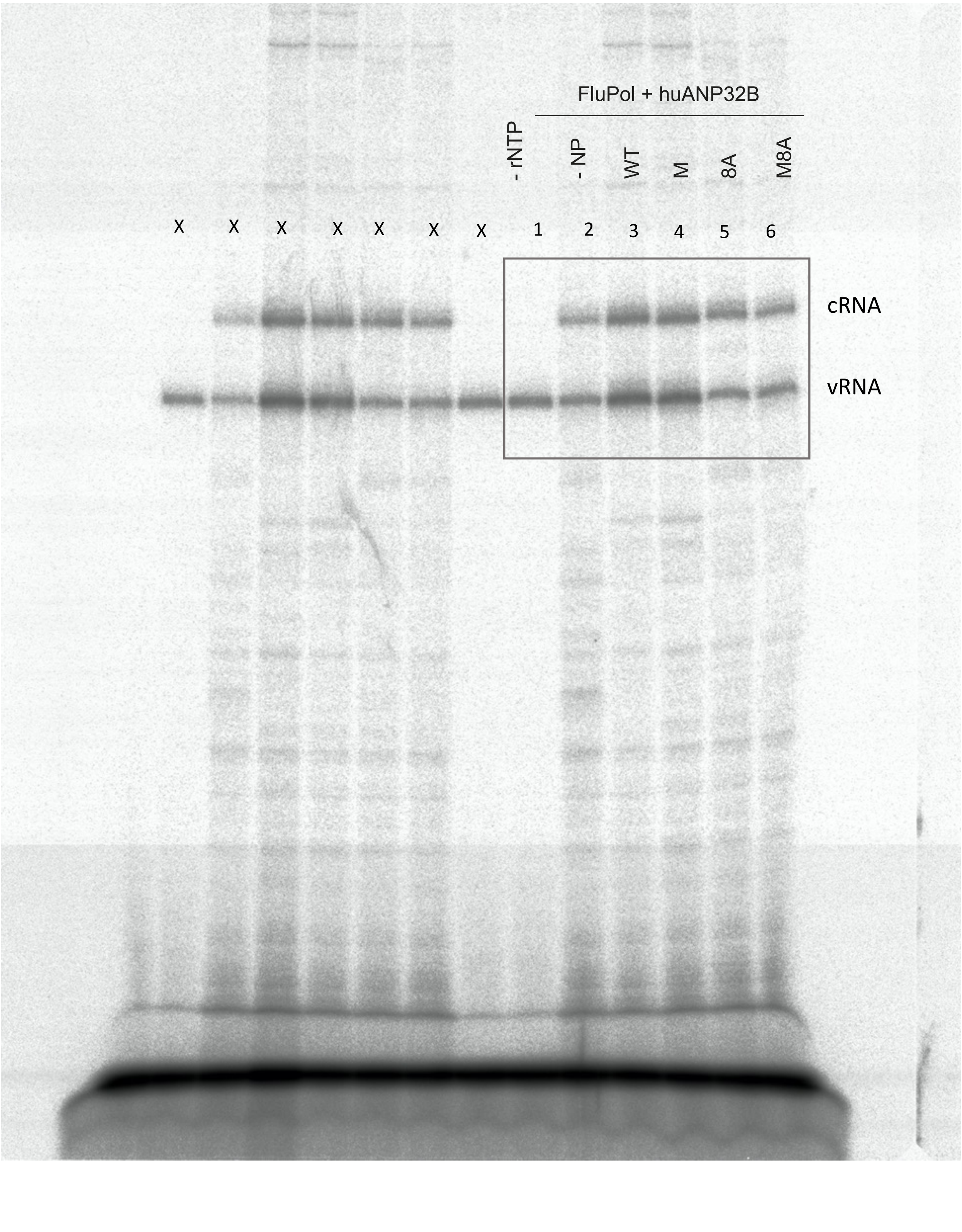

visualised by phosphorimaging on an FLA-5000 scanner

Fig 3E repeat 2

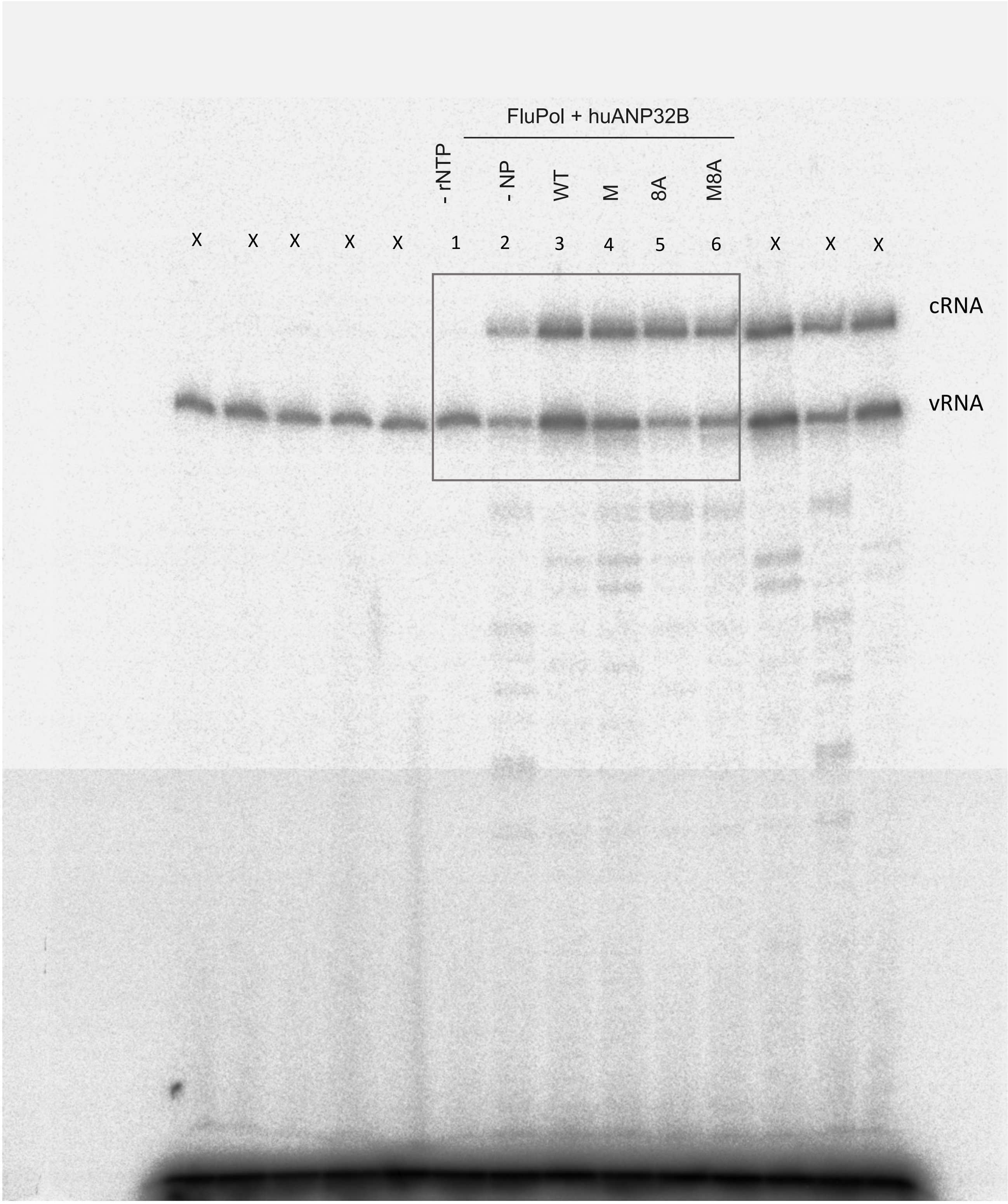

visualised by phosphorimaging on an FLA-5000 scanner

Fig 3E repeat 3

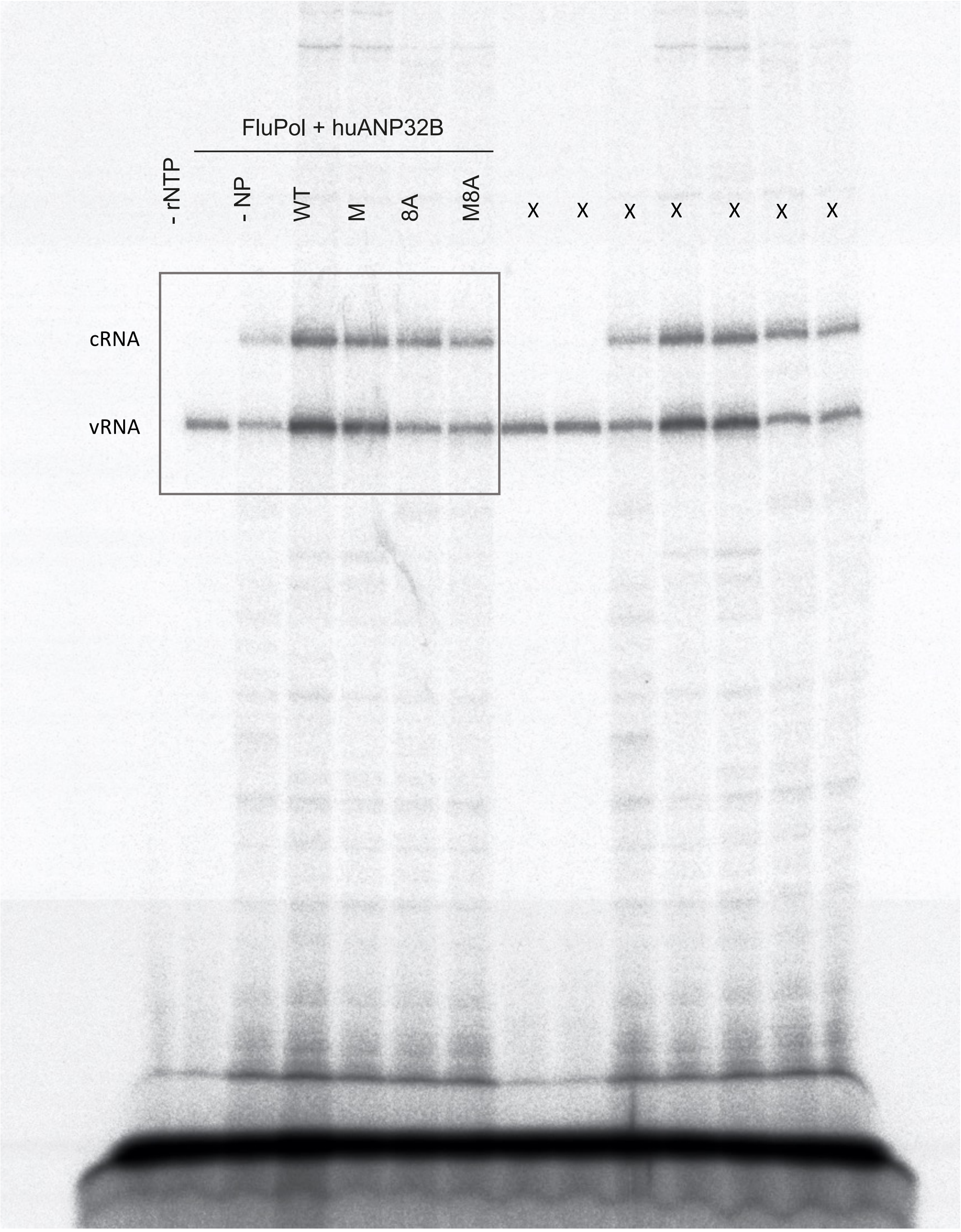

visualised by phosphorimaging on an FLA-5000 scanner

S1B Fig

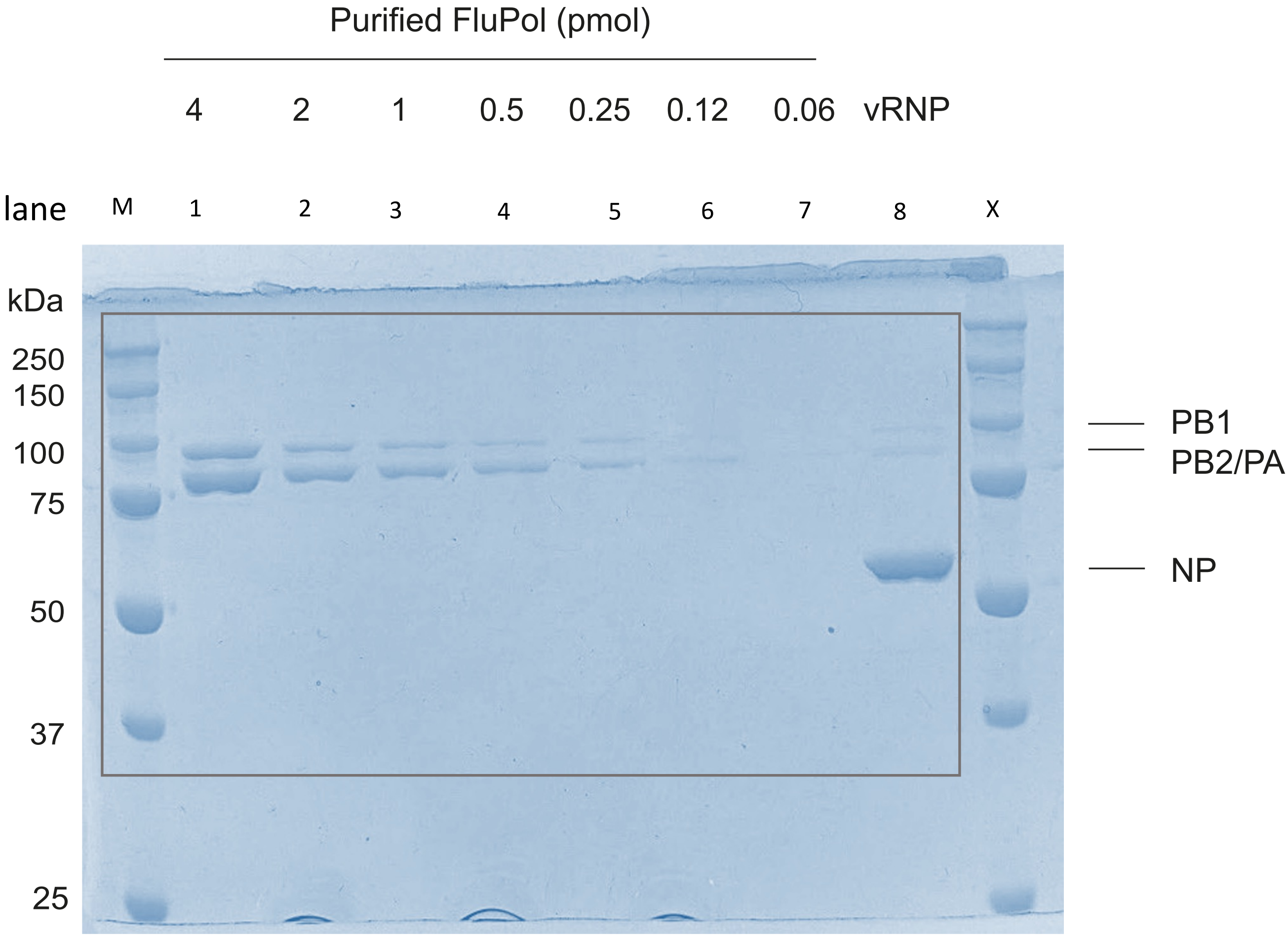

visualised and photo-captured on lightbox

S2A Fig, S2B Fig (left)

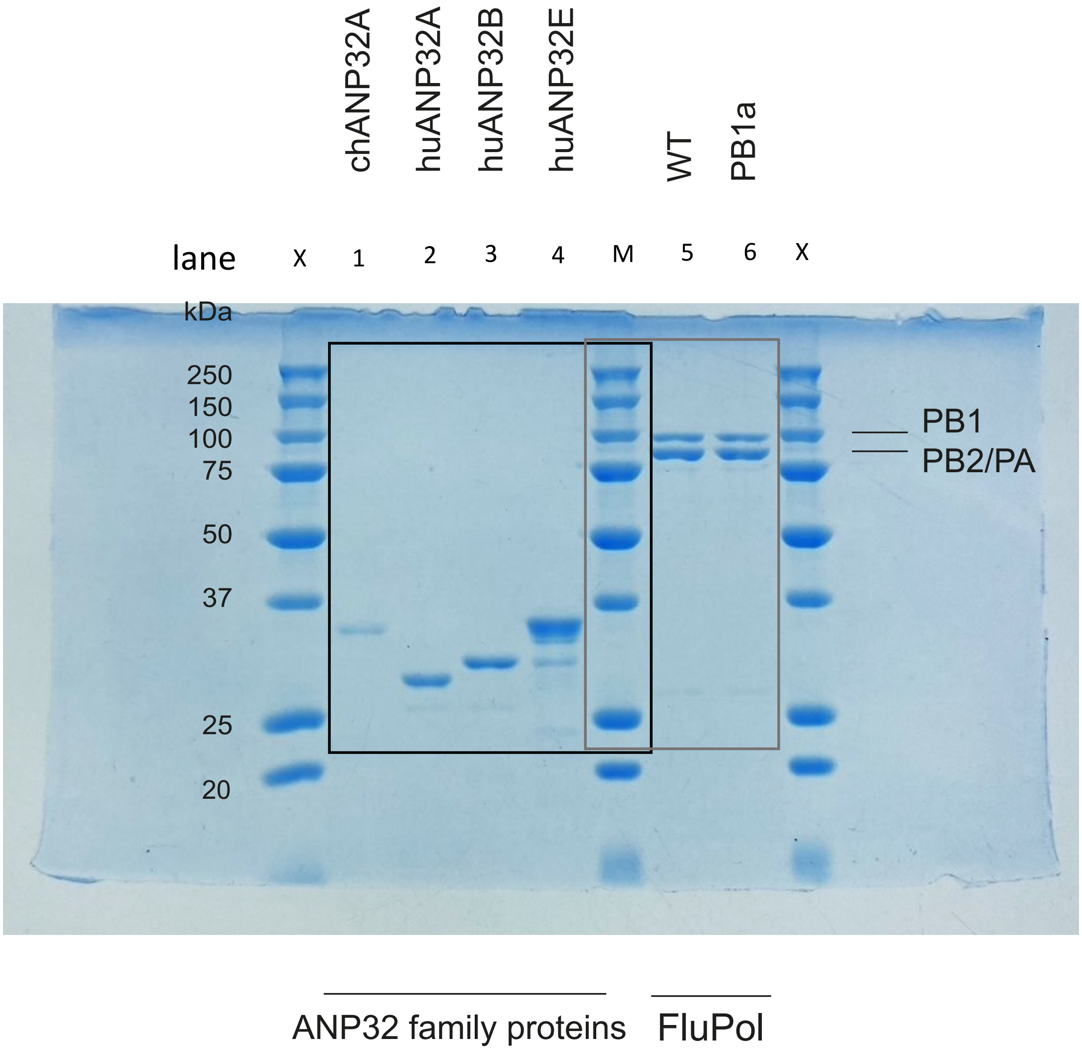

visualised and photo-captured on lightbox

S2B Fig (right)

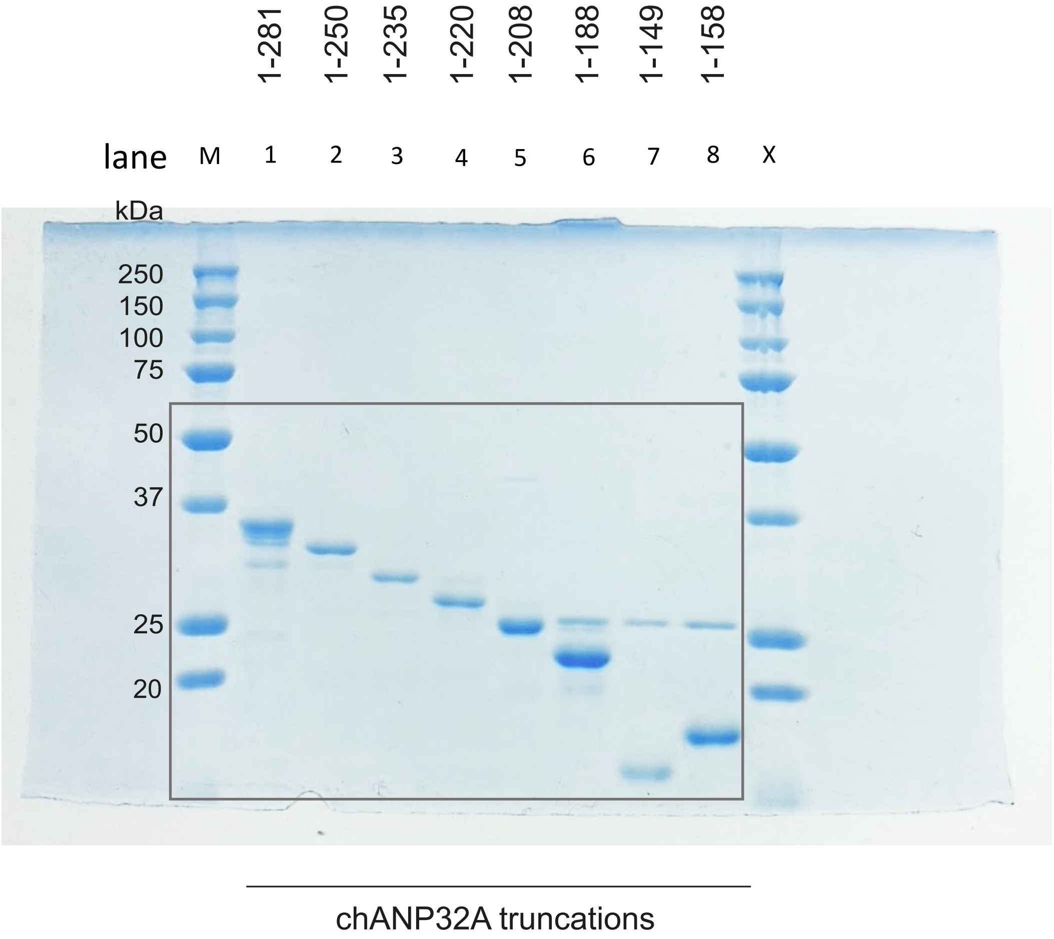

visualised and photo-captured on lightbox

S3A Fig

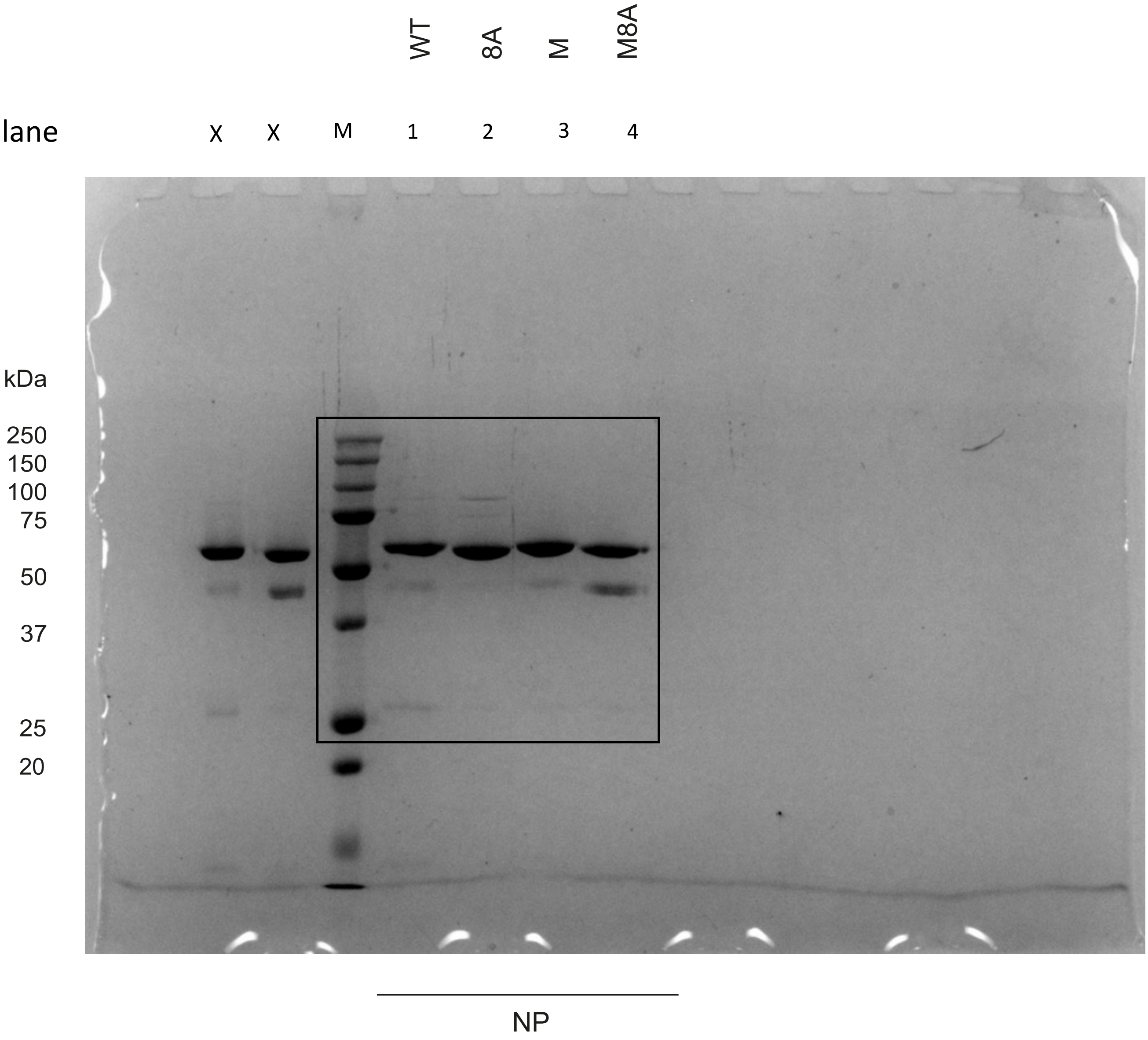

visualised and photo-captured on GelDoc

S3B Fig

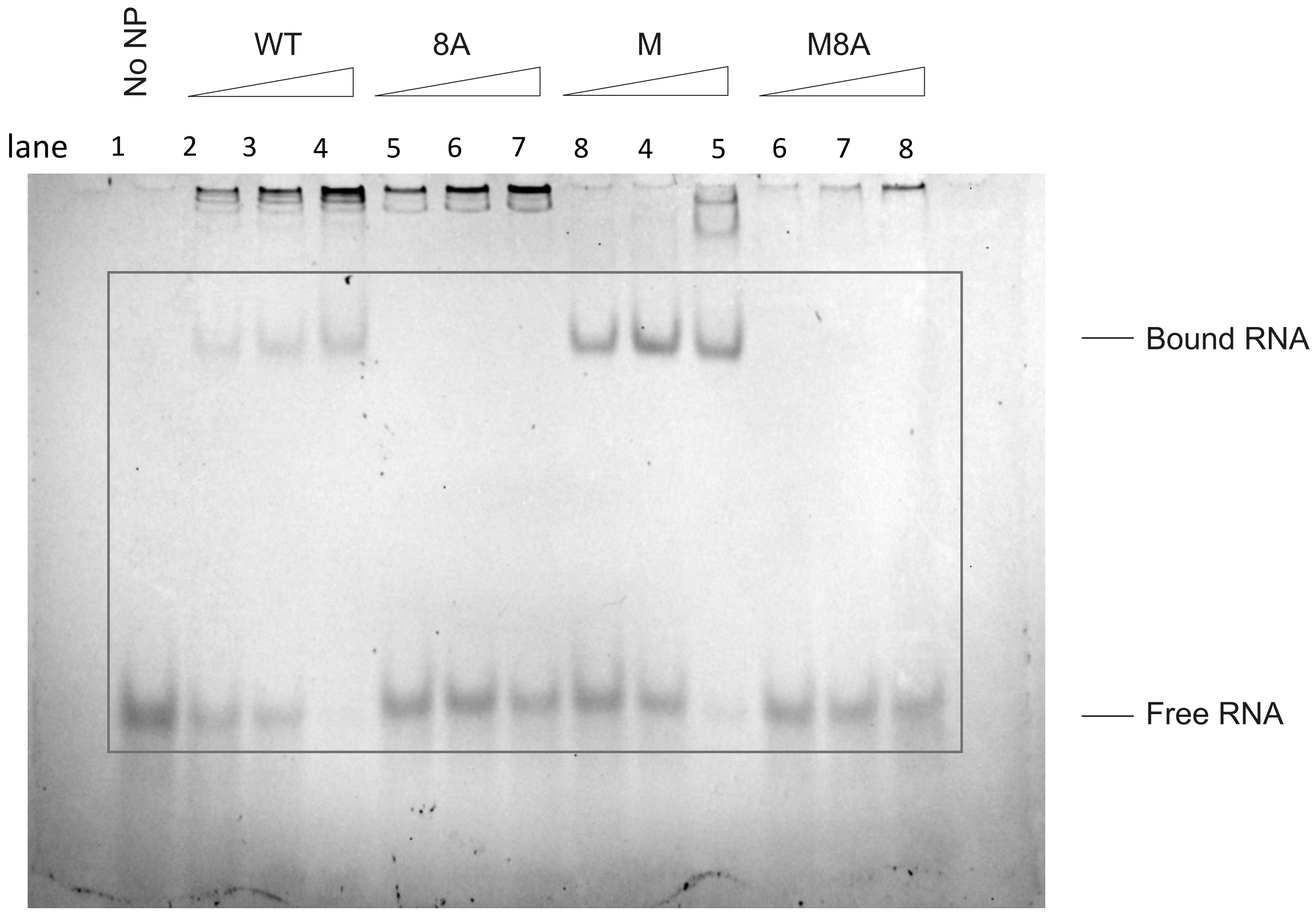

visualised and photo-captured on GelDoc

S4 Fig

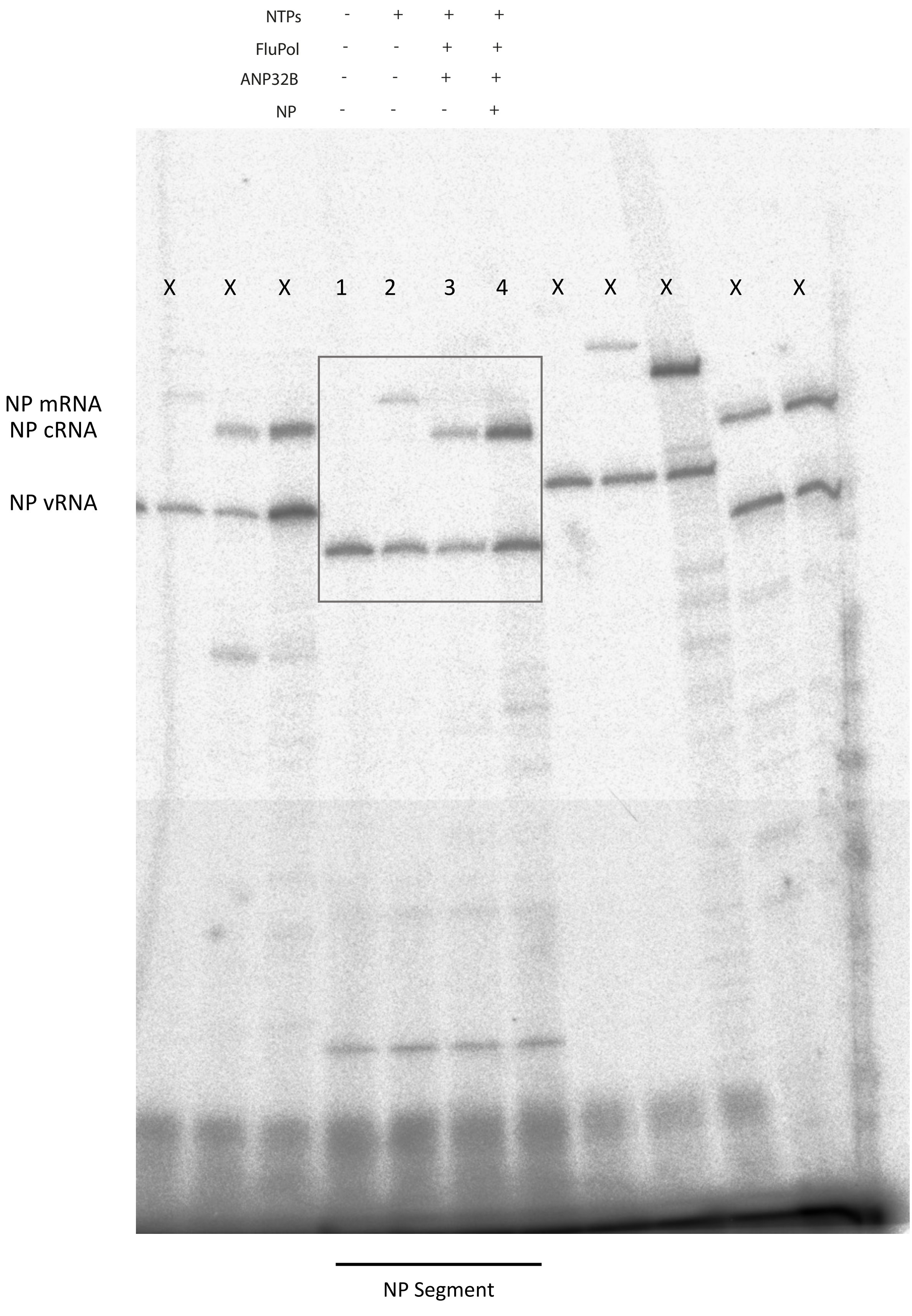

visualised by phosphorimaging on an FLA-5000 scanner

S4 Fig

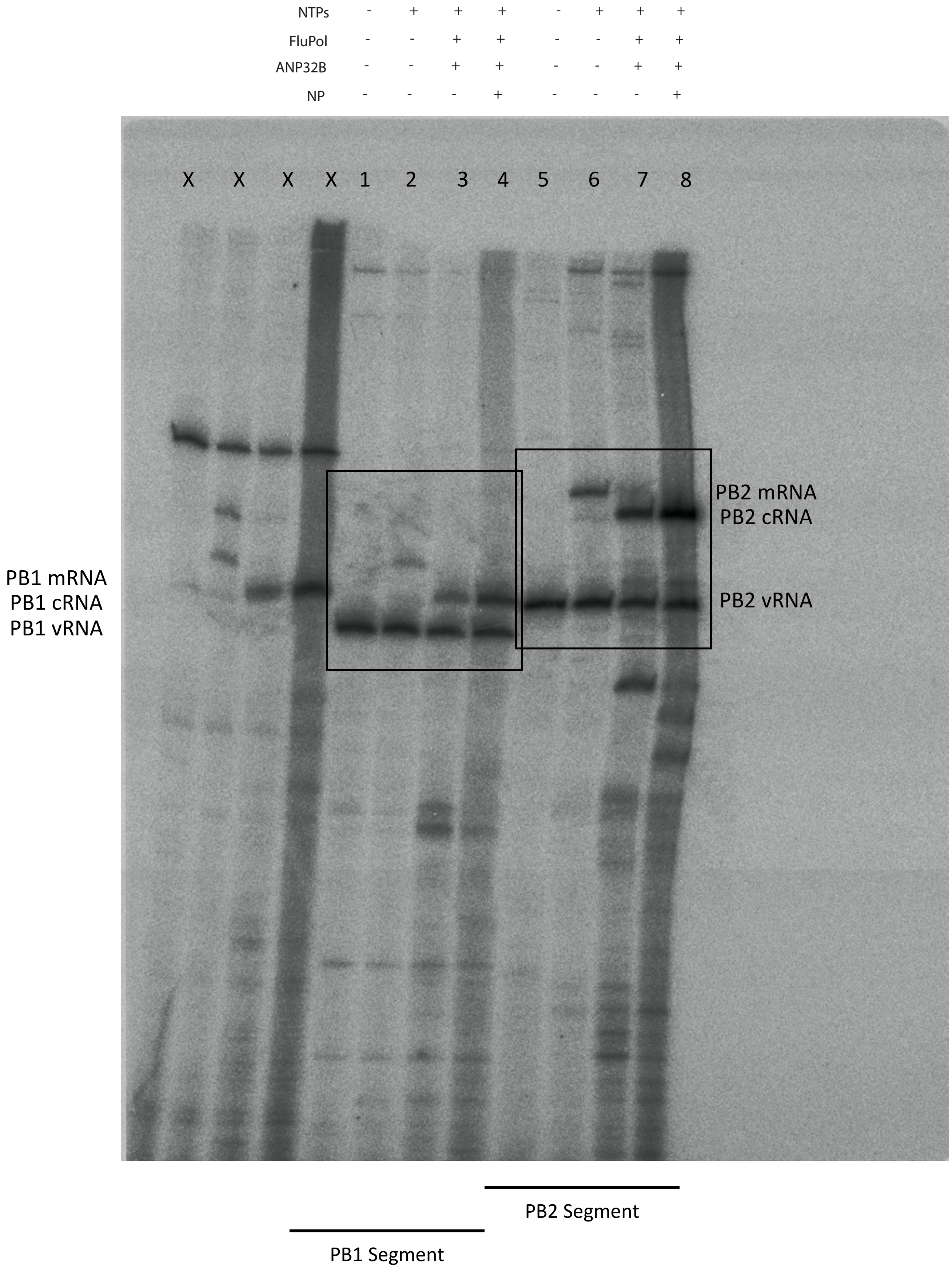

visualised by phosphorimaging on an FLA-5000 scanner

S4 Fig

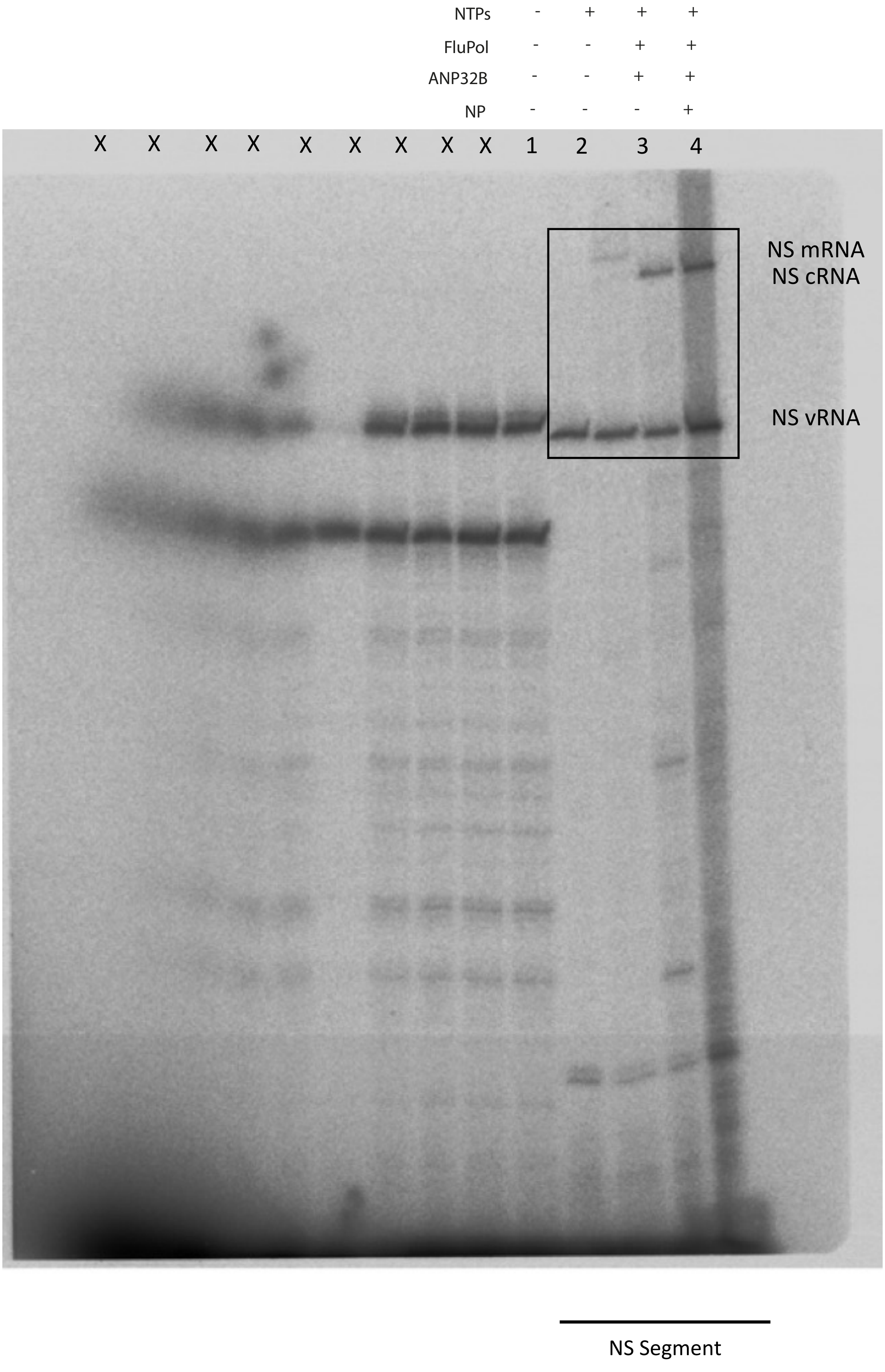

visualised by phosphorimaging on an FLA-5000 scanner
