# Supplementary material for: Defining the minimal components of the influenza A virus replication machinery via an in vitro reconstitution system
Source: PLoS Biol. 2023 Nov 9;21(11):e3002370. doi: 10.1371/journal.pbio.3002370 (PMC10662765; doi:10.1371/journal.pbio.3002370)
Supplement: Attachment — Submitted filename: Response to Reviewers Comments.docx [file pbio.3002370.s007.docx]

**Response to Reviewers’ Comments**

**Reviewer #1:**

The authors established an authentic in vitro transcription and replication system for influenza A virus, based on vRNPs isolated from virions and using recombinant viral and host factors essential for these processes. This is an substantial advancement in the research field of negative sense RNA viruses. The authors have defined the minimal components apart from vRNPs, including the host protein ANP32 as well as exogenous viral RNAP and NP. Several mechanistic issues could be clarified. Their assay provides an important platform to address many mechanistic issues of IAV transcription and replication in the future.

We thank the Reviewer for acknowledging that our work represents “a substantial advancement in the research field of negative sense RNA viruses”.

Major comments:

1) The text description of results presented in Fig. 1a, b and Fig. 3a, b and e is aggravated for the reader because the authors refer in the text only to entire part figures without referring to the specific relevant lanes. As a consequence the reader has to collect the corresponding lanes her/himself, which is a bit like 'collecting mushrooms'. For example, at first reading I did not catch the conclusion inferred from Fig. 3e, namely that the M8A variant of NP was still able to somewhat stimulate cRNA synthesis despite oligomerisation and RNA-binding deficiency.
The problem can be solved by numbering the lanes in Fig. 1a, b and Fig. 3a, b and e, and referring meticulously to the respective lanes when describing the individual results in the text.

We apologise for any confusion; we have now included lane numbers in Fig. 1a, b and Fig. 3a, b, and e and refer to the respective lanes when describing the individual results in the text.

2) Fig. 1a, b and 3a, b: make the - and + signs below the primer extension gels larger and bolder.

We have made the – and + signs bold in both figures.

3)  Page 7, lines 205/206: here it is particularly important to number the lanes in Fig. 3e and to guide the reader to the lanes showing stimulation of replication (cRNA synthesis) by NP variants 8A and M8A that lost their RNA-binding ability.

We now refer to specific lanes to help the reader.

Minor comments:

p. 4:

- line 100: "... substrates, only vRNA ..."

- line 105: "... resulted in a robust increase ..."

- line 109: "... polymerase likely promotes the replicational ..."

p. 5:

- line 112: "... we titrated increasing amounts ..."

- line 117: "... resulted in a decline of mRNA ..."

- line 133: "... among all ANP32 proteins tested, chicken ..."

- line 135: "... exhibits particularly strong binding to the viral ..."

- line 137: "... whilst human ANP32E could not, or only very weakly, promote replication."

- line 140: "... showing almost similar ...

p. 6:

- line 146: Supplementary Fig. 2d

- line 161: "... essential factor for replication of the viral genome, as only ..."

- line 175/176: "... distinct increase in vRNA level, while no such increase was observed with inactive polymerase (Fig. 3a)."

p. 7:

- line 183-185: "... further increased cRNA levels (Fig. 3c), whilst the same setup but using active viral polymerase resulted in ..."

- line 193: "... into the tail-loop ..."

- line 194: "... monomeric mutant (M, defective in NP oligomerisation), and combined these mutations to produce a monomeric NP that is also deficient in RNA binding (M8A) [23-26]. We confirmed by electrophoretic ..."

- line 196: "... that the 8A and M8A mutants are deficient ..."

- line 201: " on cRNA templates, but curiously ..."

- line 208: " The finding that an oligomerisation-defective mutant NP that retains RNA binding can ..."

p. 8:

- line 212: "... RNA binding ..."

- line 215: "... synthesis on vRNP templates in the presence ..."

- line 216: "... full cycle of replication, that is, vRNA to cRNA and cRNA to vRNA synthesis (Fig. 3f)."

p. 9:

- line 242: "... polymerase that captures the 5' end of the nascent cRNA and enables the ..."

- line 246: " These newly assembled cRNPs then give rise to vRNA synthesis, with ..."

- line 253: "... virus-infected cells ..."

- line 263: "... genome replication, with avian ..."

p. 10:

- line 288: "... double-stranded ..."

- line 290: "... such as RIG-I to trigger the activation of innate immune pathways and supress influenza ..."

- line 302: "... viral polymerase and NP are present, such ..."

p. 11:

- line 314: "... techniques to observe vRNPs at different stages during replication."

p. 14:

- line 414: "... were carried out using Prism 8."

p. 17:

- line 454: "... FluPol and/or huANP32B. In a-e, data represent mean values ± s.e.m, based on n ≥ 3 independent ..."

p. 18:

- line 465: "... and full-length or truncated ..."

- line 471: " In a and b, data represent mean values ± s.e.m, based on n ≥ 3 independent ..."

p. 21:

- line 485: "e, Effect of oligomerisation capacity and RNA-binding ability on the functions of NP in viral replication."

- line 489: "... significance. In a-e, data represent mean values ± s.e.m, based on n ≥ 3 independent ..."

p. 22:

- line 504: "... modification, from a transcriptase to a replicase that initiates cRNA synthesis in a primer-independent manner (2)."

p. 26:

- line 533: "... NP proteins were purified from ..."

- line 534: move " * indicates NP degradation product." to the legend part describing panel a (line 533); also increase the size of the asterisk in Fig. S3a.

We thank the Reviewer for their meticulous reading of our manuscript and all the minor comments; we have revised our text accordingly.

Reviewer #2:

The paper "Defining the minimal components of the influenza A virus replication machinery via an in vitro reconstitution system" by Zhu et al. is well written and of high quality. I fully agree with the authors that this study is the first to establish a fascinating in vitro method to study the different vRNP activities.

We thank the Reviewer for the positive comments.

There are some open questions that can be addressed by the authors:

1) As shown in Figure 3a and b, there is only a two-to-three-fold accumulation of vRNA in the presence of FluPol WT, NP and huANP32 but a >20 fold increase in cRNA level. Do the authors speculate that only very little functional cRNPs are generated? This should be discussed.

We agree that the differences in cRNA and vRNA production could, at least in part, be explained by the fact that not all cRNA is assembled into functional cRNPs. Additionally, it is important to note that newly synthesised cRNA and vRNA levels were expressed relative to the input cRNA and vRNA levels, respectively, and these input levels have different values. Therefore, the increase in cRNA and vRNA relative levels is not directly comparable. We included a new sentence in the Results section to address this issue.

2) Figure 3e: The monomeric NP mutant can still support cRNA/cRNP synthesis and in particular lead to an increase in vRNA levels. Though the authors quote an earlier paper regarding cRNA stabilization, however, full length vRNA accumulation seems to require NP-oligomerization (see also <https://doi.org/10.1038/ncomms2589:> e.g. Fig. 2a,). Is there an explanation for this discrepancy?

We speculate that this discrepancy can be explained by differences between the *in vitro* and cell-based assays. Specifically, cRNPs and vRNPs assembled with monomeric NP, although capable of replication, may be more susceptible to nuclease digestion in a cellular environment. To address this point, we have included two additional sentences in the Results section.

3) Has the monomeric NP mutant (R416A) from A/NT/60/1968 been shown to behave as a monomer? If not, could the authors provide biochemical data on this?

We have included new data (S3B Fig) that compare the elution profiles of the wildtype, the R416A monomeric mutant (M), the RNA-binding mutant (8A) and the monomeric RNA-binding mutant (M8A) NPs using size exclusion chromatography. Both M and M8A, which contain the R416A mutation, were found to elute as monomers, while the wildtype and 8A mutant eluted as oligomers, as expected. These new findings are consistent with previous data published by our group, demonstrating that the R416A NP mutant from A/NT/60/1968 behaves as a monomer in size exclusion chromatography (see Fig. 1a in Knight et al Acta Cryst (2021) F77, 208–214 <https://doi.org/10.1107/S2053230X2100635X>).

4) Figure 3f: The authors state that new (and functional) vRNPs are formed in the presence of FluPol WT, NP and huANP32, but there is no direct evidence for this. Can the authors rule out that NP-free vRNA accumulates? If not, this possibility should be discussed.

Thank you for highlighting this point. In the Discussion section, we erroneously referred to “functional vRNPs”, which we have now corrected to “functional cRNPs”. Additionally, we have included two new sentences in the Discussion, where we speculate that vRNA might also form functional vRNPs capable of participating in further cRNA synthesis. However, we point out that this remains to be determined, as our current assay cannot distinguish cRNA produced by *in vitro* assembled vRNPs from that synthesised by input vRNPs.

5) Lane 406: Segment 5 encodes NP and not NA.

We apologise for the error; we corrected this to segment 6 that encodes NA.

6) To show the robustness of this in vitro assay, it would be good to know the vRNP activity of a segment other than segment 5.

We have observed similar results using other segments. Specifically, we analysed the effect of FluPol, ANP32B and NP on the transcription and replication of segment 1, 2, 5 and 8 vRNAs. The new data are fully consistent with the data we observed with segment 6 vRNA. We have included these new data as S4 Fig.
